# Sex-Specific Retinal Anomalies Induced by Chronic Social Defeat Stress in Mice

**DOI:** 10.3389/fnbeh.2021.714810

**Published:** 2021-08-12

**Authors:** Eric Arsenault, Andrée-Anne Lavigne, Samaneh Mansouri, Anne-Marie Gagné, Kimberley Francis, Thibault P. Bittar, Francis Quessy, Khaled Abdallah, Annie Barbeau, Marc Hébert, Benoit Labonté

**Affiliations:** ^1^CERVO Brain Research Centre, Centre Intégré Universitaire de Santé et des Services Sociaux de la Capitale Nationale, Quebec City, QC, Canada; ^2^Department of Psychiatry and Neurosciences, Faculty of Medicine, Université Laval, Quebec City, QC, Canada; ^3^Department of Social and Preventive Medicine, Faculty of Medicine, Université Laval, Quebec City, QC, Canada; ^4^Department of Ophthalmology and Otorhinolaryngology—Head and Neck Surgery, Faculty of Medicine, Université Laval, Quebec City, QC, Canada

**Keywords:** electroretinogram (ERG), major depressive disorder (MDD), retina, sexual dimorphism, biomarker (BM), chronic social defeat stress (CSDS), stress resilience, stress susceptibility

## Abstract

Major depressive disorder (MDD) is one of the most common consequences of chronic stress. Still, there is currently no reliable biomarker to detect individuals at risk to develop the disease. Recently, the retina emerged as an effective way to investigate psychiatric disorders using the electroretinogram (ERG). In this study, cone and rod ERGs were performed in male and female C57BL/6 mice before and after chronic social defeat stress (CSDS). Mice were then divided as susceptible or resilient to stress. Our results suggest that CSDS reduces the amplitude of both oscillatory potentials and a-waves in the rods of resilient but not susceptible males. Similar effects were revealed following the analysis of the cone b-waves, which were faster after CSDS in resilient mice specifically. In females, rod ERGs revealed age-related changes with no change in cone ERGs. Finally, our analysis suggests that baseline ERG can predict with an efficacy up to 71% the expression of susceptibility and resilience before stress exposition in males and females. Overall, our findings suggest that retinal activity is a valid biomarker of stress response that could potentially serve as a tool to predict whether males and females will become susceptible or resilient when facing CSDS.

## Introduction

Affecting more than 300 million people worldwide (World Health Organization, 2018), major depressive disorder (MDD) is a leading cause of disability imposing a major burden on modern societies (Ferrari et al., 2013; World Health Organization, 2018). More than 14 to 16% of Americans will require treatment for MDD at one point in their life (Kessler et al., 2012) and only 20–40% of them will fully recover (Rush et al., 2006; Trivedi et al., 2006; Kato and Chang, 2013). Noticeably, epidemiologic studies report that women are 1.5 to 2 times more likely to develop MDD than males (Kessler et al., 1993; Angst et al., 2002), and women often exhibit more severe scores of depression associated with a higher prevalence of atypical symptoms and co-morbid anxiety (Nolen-Hoeksema, 1987; Angst et al., 2002; McLean et al., 2011). Additionally, it has been suggested that men and women respond differently to antidepressant treatment (Kornstein et al., 2000; Khan et al., 2005). While this sexual dimorphism is well recognized, the mechanisms underlying its etiology remain poorly understood.

It is well accepted that disease chronicity worsens patient prognostic. For instance, recurrence after the first MDD episode is up to 30–60% and it increases with each subsequent episode (Solomon et al., 2000; Eaton et al., 2008). Moreover, the longer the depressive symptoms are present, the harder they are to treat (Ramana et al., 1995; O’Leary et al., 2000). Therefore, early diagnostic of MDD is critical to decrease the long-term prevalence of the disease, which is why the research of an accurate biomarker tool to help clinicians diagnose depression has become increasingly prominent in the last decades. Such a tool would also benefit preclinical animal studies allowing researchers to investigate the pathology during its early development. As a matter of fact, there are still very few tools and biomarkers available for this purpose. Studies investigating the immune system in MDD patients reported various changes associated with the disease (Miller et al., 2009; Dowlati et al., 2010). Variations in serum levels of the brain-derived neurotrophic factor (BDNF) have also been associated with response to different antidepressants (Sen et al., 2008; Lopez et al., 2013) and changes in the activity of the hypothalamic-pituitary-adrenal (HPA) axis (Surget et al., 2011; Menke, 2019)—including circulating cortisol-are hallmark alterations consistently associated with the expression of the disease (Otte et al., 2016). However, the cellular complexity found in blood, along with the heterogeneity and severity of the disease, interfere with the reproducibility of these findings, especially during its prodromal phase. Consequently, there is still no reliable biomarker allowing the early detection of individuals at risk to develop MDD.

Alternatively, electroretinography (ERG) has emerged as a non-invasive and reliable approach to investigate psychiatric disorders (Lavoie et al., 2014c; Youssef et al., 2019) given that the retina belongs to the central nervous system as they share the same embryonic origin (Graw, 2010; Dowling, 2012). The ERG is a biopotential signal generated by the retina in response to a flash of light. Both rod (night vision) and cone (day vision) responses can be isolated and quantified depending on the state of retinal adaptation to darkness or light, respectively (Hebert and Lachapelle, 2003). A typical ERG waveform is composed of low-frequency waves such as the a- and b-waves, and of high-frequency waves known as the oscillatory potentials (OP). The a-wave is a negative component originating from the photoreceptors and the following b-wave is a larger positive component produced by bipolar cells (Hebert and Lachapelle, 2003). The OPs are fast oscillations superimposed on the ascending phase of the b-wave and are hypothesized to originate from the interaction between the amacrine and ganglion cells of the retina (Wachtmeister, 1998).

Interestingly, abnormal retinal activity has been consistently reported in many psychiatric disorders including MDD (Hebert et al., 2010, 2020; Fornaro et al., 2011; Fam et al., 2013). For instance, prolonged cone b-wave implicit time and reduced mixed rod/cone a- and b-wave peak amplitudes have been reported in MDD patients (Hebert et al., 2017). Interestingly, the cone b-wave delay identified in MDD patients was similar to the anomalies found in schizophrenia (SZ; Hebert et al., 2015) and bipolar disorder (BP) patients (Hebert et al., 2020) whereas the mixed rod/cone alterations were common to BP, SZ and children at high risk to develop SZ or BP (Hebert et al., 2010). Prolonged cone b-wave implicit times were also reported in MDD patients with suicidal ideation or melancholic anhedonia (Fountoulakis et al., 2005) hence highlighting this ERG alteration as a major manifestation of psychiatric disorders including MDD. In seasonal affective disorder-an MDD-related disease characterized by atypical symptoms-affected patients demonstrated a decreased rod sensitivity in winter months when compared to healthy controls (Hebert et al., 2004; Lavoie et al., 2009). Noticeably, the degree of change in the rod sensitivity was directly associated with the severity of the depressive symptoms (Lavoie et al., 2009, 2014c). Interestingly, these alterations were normalized during the remission in summer months or after 4 weeks of light therapy (Lavoie et al., 2009) suggesting the ERG anomalies could represent a state marker of the disease. Likewise, MDD patients responding to Duloxetine, a serotonin-norepinephrine reuptake inhibitor (SNRI) antidepressant treatment, exhibited higher rod b-wave amplitudes than non-responders before treatment (Fornaro et al., 2011) suggesting that ERG could also be used to predict treatment response. Sexual dimorphism was also examined in clinical and pre-clinical studies on ERG. For instance, lower mixed rod/cone b-wave amplitudes were reported in women suffering from seasonal affective disorder when compared to healthy women whereas the opposite effect was displayed in men (Lam et al., 1992). In a preclinical study on SZ, male transgenic mice having *N*-methyl-*D*-aspartate receptor (NMDAR) hypofunction resulting from D-Serine deficiency (SR^−^/^−^) exhibited longer mixed rod/cone b-wave implicit times than their female counterparts. Furthermore, in the wild-type mice, males showed higher mixed rod/cone b-wave amplitudes than females (Torres Jimenez et al., 2020). Altogether, these findings suggest that ERG may be sensitive to sexual dimorphic features in both humans and mice psychiatric conditions. Finally, alterations have also been reported in other models useful for the study of depressive-like and psychosis-like behaviors in mice (e.g., Lavoie et al., 2014a,b, 2015). For instance, a decreased rod retinal sensitivity was observed in mice that had five times more brain dopamine (DA) content (dopamine transporter knockout mice; DAT-KO) than wild-type mice. Similarly, the cone b-wave implicit time was prolonged in mice that had a reduction of the brain serotonin (5-HT) synthesis (tryptophan hydroxylase 2 knockin mice; Tph2-KI) whereas a decreased cone amplitude and the sum of cone OPs was observed in mice missing D1 receptors in the striatum (dopamine receptor D_1_ knockout mice; D1R-KO). These ERG anomalies were found whereas no changes in the mice’s retinal monoamine content were observed (Lavoie et al., 2014b). Together, these results suggest that ERG signals in mice can be affected by central brain neuromodulation of DA and 5-HT neurotransmitters-known to have key roles in various psychiatric disorders (Meyer et al., 2001; Keeney et al., 2006; Sarchiapone et al., 2006; Belmaker and Agam, 2008; Saini et al., 2008; Watt et al., 2009; Viikki et al., 2010; Tse et al., 2014; Sachs et al., 2015; Iniguez et al., 2016; Favoretto et al., 2020)—and provide interesting insights into the etiological mechanisms underlying stress response in males and females.

In this study, we tested whether chronic social stress impacts retinal activity in male and female mice and reproduces the retinal alterations observed in MDD patients. To do so, we used a model of chronic social defeat stress (CSDS) known to induce behavioral features relevant to the clinical manifestations of MDD in men and women (Nestler and Hyman, 2010). The CSDS involves subjecting mice to repeated bouts of social subordination, after which a subset of mice exhibits depressive-like behaviors defined by anhedonia, anxiety-like responses, and social withdrawal (Golden et al., 2011; Harris et al., 2018). Interestingly, CSDS in males and females also allows differentiating a subpopulation of resilient mice resistant to the anhedonia and social deficits seen in susceptible mice (Krishnan et al., 2007; Iniguez et al., 2014, 2018). We used ERG to test whether changes in rod and cone activity associate with the expression of susceptibility and resilience to CSDS in males and females and whether ERG signals before stress exposure could predict stress responses in both sexes.

## Materials and Methods

### Animals

Male (*n* = 49) and female (*n* = 41) C57BL/6NCrl (C57BL/6) mice aged 8 weeks old and male Crl:CD1(ICR; CD1) mice aged 4–6 months old were obtained from Charles River Laboratories (USA). At baseline, the male and female C57BL/6 mice had body weights of 22.9 ± 1.1 g and 20.2 ± 1.4 g, respectively (mean ± SD). All mice were fed ad libitum at 22–25°C on a 12-h light/dark cycle. The CD1 mice were singly housed except during the social defeat procedure. The C57BL/6 mice were housed in groups (four per cage) for 5 days of acclimatization before and singly housed after the CSDS. The 90 C57BL/6 mice were randomly divided into four groups: 35 males and 28 females were subjected to CSDS (defeated groups) and 14 males and 13 females served as controls (control groups). All experimental procedures were approved by Université Laval’s Institutional Animal Care Committee in respect of the Canadian Council on Animal Care guidelines.

### Chronic Social Defeat Stress

CSDS was performed in males (Golden et al., 2011) and females (Harris et al., 2018) as described before. Briefly, in males, C57BL/6 mice were first introduced to unknown aggressive retired CD1 breeders in their home cages for a period of 5 min during which the C57BL/6 intruders were attacked by the resident mice. Following this initial phase, C57BL/6 mice were housed to the other side of the cages-which were divided in half by perforated acrylic plates-for 24 h allowing continuous sensory contact with the CD1 mice without physical harm. The same procedure was repeated over 10 days with each C57BL/6 mouse meeting a new unknown CD1 mouse every day.

To ensure that all C57BL/6 mice received an equivalent number of attacks each day, each CD1 mouse was initially screened for aggressive behavior prior to the stress paradigm. The screening took place on the 3 days preceding the first defeat during which the CD1 mice attacked C57BL/6 screeners for 3 min each day. Only the aggressors attacking rapidly the screening mice daily were selected for defeat. Regarding the controls, C57BL/6 mice were housed two per cage separated by a perforated acrylic divider and housed in the same room as the mice undergoing CSDS. Sawdust was used as litter in both defeat and control cages.

In females, a similar approach was used with the difference that the base of the tail and the pubis of the C57BL/6 stressed mice were soaked with 50 μl of urine from an unknown CD1 male mouse before each defeat bout. The 5-minute social stress bouts were interrupted every time the CD1 male mice mounted the C57BL/6 females. All C57BL/6 female mice were associated with a different male urine so that aggressor CD1 mice never encountered the same urine twice. Urine collection was performed during the week preceding social defeat stress with metabolic cages (Tecniplast Group, Tecniplast Canada, Canada). To do so, retired CD1 breeders were housed in the metabolic cages overnight and urine was collected each morning and stored at 4°C after being filtered. Although there are subtle differences between the methodology in males and females, both approaches are comparable inducing similar depressive- and anxiety-like symptoms along with subpopulations of susceptible and resilient mice (Harris et al., 2018).

Social-avoidance behavior was assessed with the social interaction (SI) test 24 h after the end of the social defeat paradigm as described before (Golden et al., 2011; Harris et al., 2018). Briefly, the SI test consisted of two phases of 150 s each taking place in a large arena with three designated areas: (1) a social interaction zone on one side surrounding a mesh-wire enclosure; (2) the two opposite corners from the social interaction zone; and (3) the open field which is the remaining space of the arena. In the first phase of the test, C57BL/6 mice explored the arena with no target CD1 aggressor in the social interaction zone. This initial phase was followed by a second exploratory phase but this time in the presence of an unknown target CD1 aggressor maintained into the enclosure within the social interaction zone. Time spent in the different zones of the arena was automatically recorded through ANY-Maze Video Tracking Software 4.99 (Stoelting Co., Wood Dale, IL, USA) using a top-view camera, therefore providing unbiased behavioral analyses. Defeated mice were then designated as susceptible or resilient based on their social interaction (SI) ratio (time in interaction zone with social target / time in interaction zone without social target): mice with a ratio <1.0 were phenotyped as susceptible and >1.0 as resilient. This measure of susceptibility vs. resilience has been shown to correlate with other defeat-induced behavioral abnormalities such as anhedonia and increased sensitivity to inescapable stresses (Krishnan et al., 2007; Iniguez et al., 2014, 2018). Defeats and SI tests both were performed in two different rooms during the light cycle of the animals. While the CSDS took place in a bright room, the SI tests were in a dark room under a dimmed red light.

### Electroretinography Recordings

The ERGs were performed on the C57BL/6 mice right after their 12 h dark cycle. All mice were anesthetized with an intraperitoneal injection of a Ketamine (100 mg/Kg) and Xylazine (10 mg/Kg) mixture under dim red light and a booster dose of Ketamine (50 mg/Kg) was administered between the scotopic and photopic ERGs. The left eye cornea was anesthetized with 0.5% proparacaine hydrochloride (Alcaine, Alcon Canada, Canada) and the pupil was dilated with 1% tropicamide (Mydriacyl, Alcon Canada, Canada) 10 min prior to testing. Lubricant eye drops (Systane Gel Drops, Alcon Canada, Canada) were used to prevent dryness of the cornea of both eyes. A loop-shaped DTL electrode (Shieldex 33/9 Thread, Statex, Germany) was placed directly on the left eye cornea to record ERG signals. Reference and ground subcutaneous electrodes (Grass Technologies, Astro-Med, Canada) were placed respectively on the forehead and tail of the animals. During the recordings, mice were lying down on a homeothermic blanket (Harvard Apparatus, Holliston, MA, USA) to maintain body temperature around 37°C.

For every animal, rods’ function was measured in the dark (scotopic condition) followed by cones’ function measured under light (photopic condition) using an Espion E1 system with flash stimulation provided by a Ganzfeld ColorDome (Diagnosys LLC, Lowell, MA, USA). The ERG signals were recorded using the Espion Acquisition Software version 3.0.1 (Diagnosys LLC, Lowell, MA, USA). Prior to the scotopic ERG, mice’s eyes were adapted to dark for 1 h, which is sufficient for rods to recover from a complete bleach in both humans and mice (Kang Derwent et al., 2002; Cameron et al., 2006). A light adaptation period of 10 min at 30 cd/m^2^ was allowed before photopic recordings. Rods’ function was assessed using seven white flashes of luminance increasing from −0.020 to 2.859 log cd.s/m^2^ produced by the Color Dome light-emitting diodes (LED). Interstimulus intervals were 15 s for the first recorded luminance step and 30 s for the last six brightest luminance steps. Color Dome xenon flashes were used for photopic ERG. In this condition, five increasing flashes ranging from 0.885 to 2.859 log cd.s/m^2^ were used with interstimulus intervals set at 30 s. For both scotopic and photopic ERG, at least four responses were recorded for each flash stimulation.

**Figure 1:**
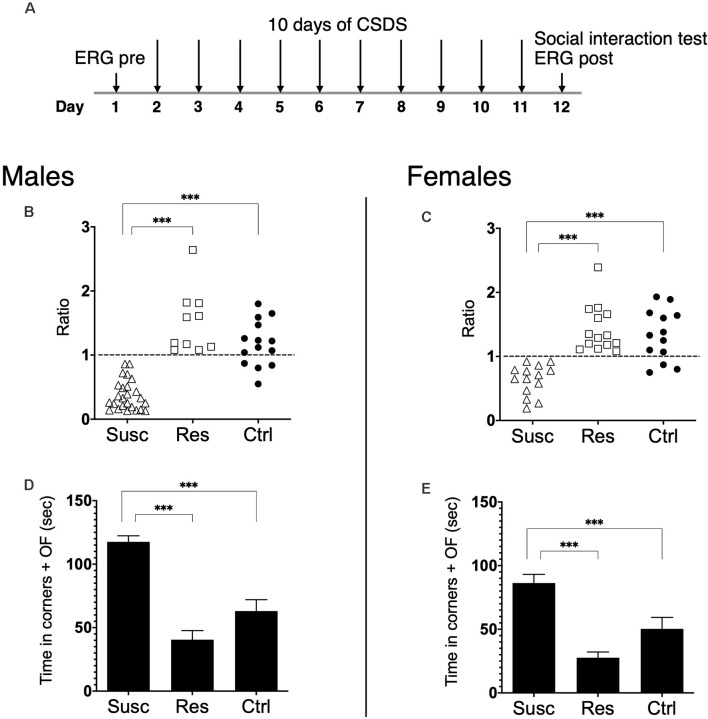
**(A)** Schematic representation of the experimental timeline. Pre-stress ERG was assessed at day 1, followed by 10 days of CSDS 24 h after (day 2, ending day 11). The social interaction test and the post-stress ERG were assessed 24 h after the last defeat on day 12. **(B,C)** Susceptible, resilient, and control mice are divided according to their SI ratio score. Male **(B)** and female **(C)** mice susceptible to CSDS exhibit social avoidance (ratio <1) when compared to male and female resilient and control mice. **(D,E)** Male **(D)** and female **(E)** susceptible mice spent significantly more time in the corners and the open field (OF) areas than the male and female resilient and control mice. Significance was determined using two-way ANOVA with Bonferroni multiple comparisons test. Symbols and bars represent the group average ± SEM; ***p < 0.001; *n*-values are given in the “Materials and Methods” section.

Baseline ERG measures were collected for every mouse one day before the beginning of the defeat stress and repeated 24 h after the last defeat (Figure 1A). To determine estrous cycles for every female mouse, vaginal smears were collected before the pre-stress ERG recording and at post-stress before the SI test.

### Data Analysis

The SI ratios of the mice were presented as mean and standard error for each phenotype (susceptible, resilient, and control). After checking for the homogeneity of variance and normality of the data set, a two-way ANOVA was used to compare the social interaction (SI) ratios of each sex and phenotype to test whether susceptible mice interacted less with the target CD1 than the resilient and control mice. Post hoc were analyzed using Bonferroni multiple comparisons test. As for the difference between the three phenotypes in avoiding the target CD1, the time spent in the corners and the open field was compared to the time spent in the interaction zone during the second phase of the SI test using the same analysis model. In order to investigate if the estrous cycle had repercussions on the phenotype of the female, a Kruskal-Wallis test was used comparing the SI ratios of the females according to their estrous phase (pro-estrus, estrus, metestrus, diestrus) measured the same day as the social interaction test. *Post hoc* were analyzed using Dunn’s multiple comparisons test. The SI ratios of the reproductive (pro-estrus and estrus) vs. non-reproductive (metestrus and diestrus) days of the estrous cycle were also compared using a Mann-Whitney test.

**Figure 2:**
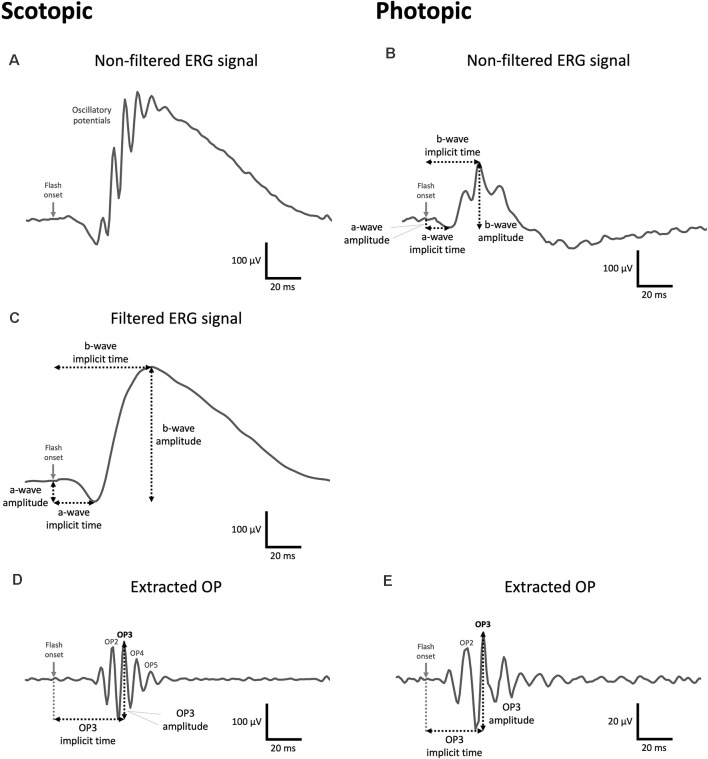
Examples of a control mouse ERG waveforms obtained at 1.37 log cd.s/m^2^ under photopic and scotopic conditions. **(A,B)** Example of a raw scotopic **(A)** and photopic **(B)** signal displaying OPs on the rising phase of the b-wave. **(C)** Every scotopic signal was filtered (digital band-stop: 75–300 Hz) prior to the identification of the a- and b-waves. **(B,C)** a- and b-wave amplitudes are calculated (in microvolts) from the baseline to the trough of the a-wave and, from there, to the peak of the b-wave, respectively. The implicit time corresponds to the total time (in milliseconds) from the flash onset until the trough of the a-wave or the peak of the b-wave are reached, respectively. Note in the scotopic condition that ERG components were obtained from filtered signals. **(D,E)** Scotopic **(D)** and photopic **(E)** OPs were extracted from raw signals by applying digital band-pass filtering (75–300 Hz). The OP amplitude is measured (in microvolts) from the peak of the OP to the preceding trough, while the implicit time (in milliseconds) is the total amount of time from the flash onset to the peak of the OP. The measurement of the amplitude and implicit time in OP3 is shown as an example in **(D)** and **(E)** but both ERG components were calculated for each individual OP. Note the vertical scale bar represents 20 μV in **(E)**.

Detailed ERG analyses are provided in Supplementary Material and examples of typical ERG waveforms exhibiting featured waves and measured components are presented in Figure 2. Briefly, to remove the OP contamination from the a- and b-wave measurements, low-frequency waves were isolated from the average scotopic signals applying a band-stop filter from 75 to 300 Hz (Figure 2C). Conversely, OPs were extracted from both scotopic and photopic ERGs (Figures 2D,E) by removing the low frequencies applying digital band-pass filtering from 75 to 300 Hz (Lavoie et al., 2014b; McCulloch et al., 2015). Amplitudes and implicit times of the OPs and a- and b-waves were measured for each resultant wave using OriginPro 2020 (OriginLab Corp., Northampton, MA, USA). Note that mice were identified by identifier (ID) numbers during all ERG recordings as well as during the signal processing and assessment in order to generate unbiased data. Statistical analyses were performed at every luminance step on the total OP amplitudes (summation of the peak amplitude measured in the first four OPs and first two OPs in scotopic and photopic conditions, respectively) and the a- and b-wave amplitudes and implicit times with SPSS Statistics 27.0 (IBM Corp., Armonk, NY). For both scotopic and photopic ERGs, a linear mixed model analysis was performed to compare male and female susceptible, resilient, and control mice at baseline using the recorded luminance step (scotopic: −0.020–2.859 log cd.s/m^2^; photopic: 0.885–2.859 log cd.s/m^2^) as a covariate. Post hoc tests were performed using Fisher’s least significant difference (LSD) multiple comparisons test. Variations in ERG components for each sex were then tested separately using linear mixed model analyses in which phenotype (susceptible, resilient, and control) and time of assessment (pre-stress vs. post-stress) were included as main factors. LSD multiple comparisons test was used for *post hoc* analyses. All *p*-values under 0.05 were considered as significant.

Multiple backward logistic regressions were used to obtain the best combination of ERG components with sex as covariates to predict post-stress phenotype in male and female mice. Males and females datasets were pooled to increase the sample size for these analyses. Scotopic and photopic ERGs were tested separately. The accuracy of the model was calculated with the area under the receiver operating characteristic (AU-ROC) curves: an AU-ROC curve of 1 corresponded to a model with perfect discrimination ability while an AU-ROC of 0.5 corresponded to a model with no discrimination ability (meaning that the probability of having the predicted phenotype would equal chance). Sensitivity and specificity were estimated by comparing the predicted group of each mouse to their true group. In order to promote optimal predictions, all ERG components obtained from low- (e.g., a-wave amplitude and implicit time) and high-frequency (e.g., individual OP amplitude and implicit time) signals were used for this analysis. The multiple backward logistic regression analyses were performed with SAS/STAT (SAS 9.4; SAS Corp., Cary, NC, USA).

## Results

In this study, we performed scotopic and photopic ERGs before (pre) and after (post) a CSDS in male and female mice (Figure 1A). The control mice ERGs were also assessed during the pre- and post-stress periods without being submitted to CSDS. Under this experimental design, we tested with the ERG: (1) whether susceptible and resilient mice exhibited specific changes in the activity of retinal cells; (2) whether social stress changes retinal cells activity differently in males and females; and (3) whether measures at baseline (pre-stress) could predict which male and female mice would become susceptible or resilient to social defeat stress.

First, our results show that CSDS induced the expression of stress susceptibility and resilience in both males and females. Precisely, our analysis revealed a main effect of phenotype (*F*_(2,87)_ = 56.0, *p* < 0.001) highlighting that 10 days of CSDS induced social avoidance (SI ratio < 1) in 25 males and 14 females while the remaining 10 males and 14 females continued to interact with CD1 targets (Figures 1B,C). Noticeably, the two-way ANOVA revealed neither the sex by phenotype interaction (*F*_(2,87)_ = 1.87, *p* > 0.05) nor the main effect of sex (*F*_(1,87)_ = 1.36, *p* > 0.05) suggesting CSDS had the same impact in both males and females inducing subpopulations of susceptible and resilient mice. As expected, susceptible mice rather spent significantly more time in the corners and the open field avoiding CD1 targets compared to control and resilient mice in both sexes (main effect of phenotype: *F*_(2,87)_ = 52.6, *p* < 0.001; Figures 1D,E). Furthermore, our analysis detected no effect of the estrous cycle on SI ratios when comparing by estrous cycles (χ*2* = 1.38, *p* = 0.711, *df* = 3) or by reproductive days (*U* = 180.5, *p* = 0.703, *r* = −0.06 ; Supplementary Table 1).

## Sex-Specific Retinal Activity Measured With the Electroretinography

**Figure 3:**
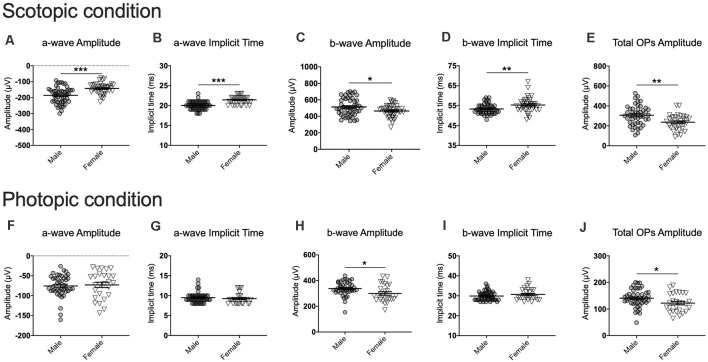
Examples of comparison of males and females for each ERG component at baseline for the scotopic (1.89 log cd.s/m^2^; **A–E**) and photopic (2.86 log cd.s/m^2^; **F–J**) conditions. Note that all luminance steps were included in the analyses although they are not all presented here. **(A–J)** Overall, males exhibited stronger and faster signals than females at baseline. Significance was determined using linear mixed model analysis with LSD multiple comparisons test. Scatter plots depict each sample value of all groups of mice and bars represent the group average ± SEM; *p < 0.05, **p < 0.01, ***p < 0.001; *n*-values are given in the “Materials and Methods” section.

One of the main objectives of this study was to test whether photopic and scotopic retinal activities vary in males and females. To answer this question, we first used a linear mixed model analysis comparing the baseline ERGs of the susceptible, resilient, and control male and female mice. Overall, for most ERG components, our analyses revealed a significant sex by phenotype interaction with *post hoc* analyses suggesting that males have higher amplitudes and faster implicit times than females. This was true in both scotopic (a-wave amplitude: *F*_(5,108.31)_ = 3.05, *p* < 0.05; a-wave implicit time: *F*_(5,183.44)_ = 7.77, *p* < 0.001; b-wave amplitude: *F*_(5,357.04)_ = 6.30, *p* < 0.001; b-wave implicit time: *F*_(5,273.31)_ = 5.38, *p* < 0.001; total OP amplitude: *F*_(5,349.07)_ = 10.85, *p* < 0.001; Figures 3A–E) and photopic conditions (a-wave amplitude: *F*_(5,179.61)_ = 2.95, *p* < 0.05; a-wave implicit time: *F*_(5,223.12)_ = 0.94, *p* > 0.05; b-wave amplitude: *F*_(5,175.29)_ = 6.05, *p* < 0.001; b-wave implicit time: *F*_(5,297,18)_ = 2.75, *p* < 0.05; total OP amplitude: *F*_(5,109.97)_ = 5.44, *p* < 0.001; Figures 3F–J). Because of this important sexual dimorphism, male and female ERG activity was analyzed separately.

## Variation in Retinal Activity of Mice Becoming Susceptible or Resilient to Social Defeat Stress

We next tested the impact of CSDS on ERGs by using a two-way mixed model ANOVA with time (pre- vs. post-stress) and mouse phenotype (control, susceptible and resilient) as main factors. For both scotopic and photopic conditions, we examined the impact of CSDS on the signals at every luminance tested. Our analyses revealed a major impact of CSDS at several luminance steps with predominant effects at 1.89 log cd.s/m^2^ in scotopic and 2.86 log cd.s/m^2^ in photopic conditions, respectively. Consequently, the following sections concentrate on these respective luminance steps although results from all recorded luminance steps are presented as a resource in Supplementary Tables 2–13.

### Scotopic Condition (Rod ERG)

**Figure 4:**
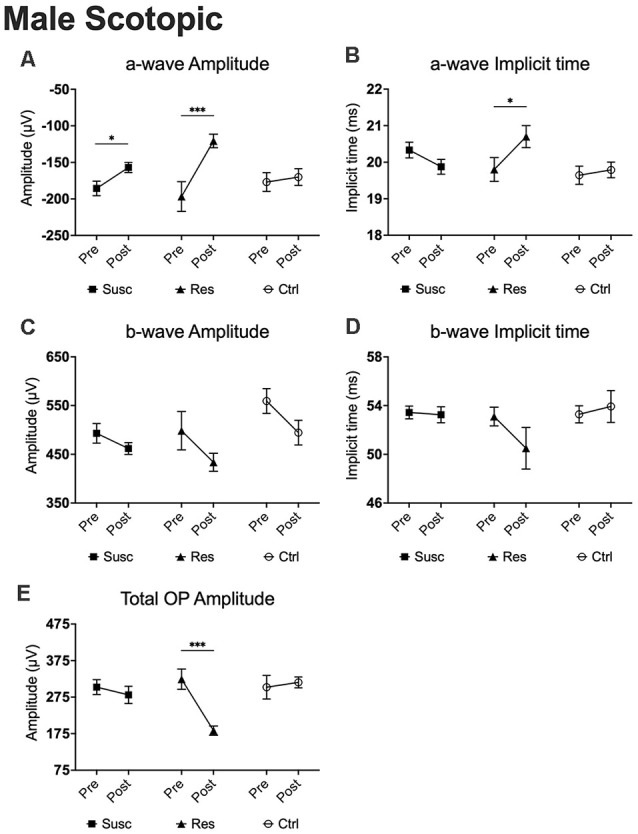
The evolution of each ERG component under the scotopic condition at baseline and after CSDS in male mice representing the susceptible, resilient, and control male a-wave amplitude **(A)**, a-wave implicit time **(B)**, b-wave amplitude **(C)**, b-wave implicit time **(D)**, and total OP amplitude **(E)**. Significance was determined using linear mixed model analysis with LSD multiple comparisons test. Symbols and bars represent the group average ± SEM; *p < 0.05, ***p < 0.001; *n*-values are given in the “Materials and Methods” section.

**Figure 5:**
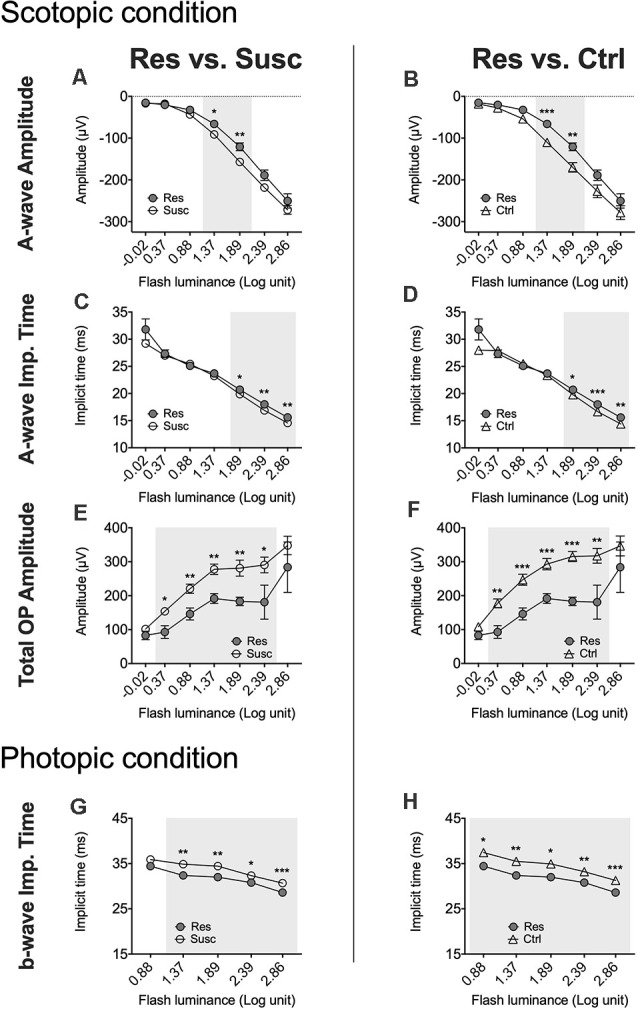
Luminance response functions comparing the post-stress ERGs of the resilient vs. susceptible, and the resilient vs. control mice in males throughout every luminance step measured in scotopic (−0.02–2.86 log cd.s/m^2^) and photopic (0.88 and 2.86 log cd.s/m^2^) conditions. **(A–H)** Resilience-specific alterations were consistently found in rod a-wave amplitude **(A,B)** and implicit time **(C,D)**, in total rod OP amplitude **(E,F)** and cone b-wave implicit time **(G,H)** following CSDS. Significance was determined using linear mixed model analysis with LSD multiple comparisons test. Symbols and bars represent the group average ± SEM; *p < 0.05, **p < 0.01, ***p < 0.001; *n*-values are given in the “Materials and Methods” section.

In males, analysis of the a-wave amplitude (1.89 log cd.s/m^2^) revealed a significant time by phenotype interaction (*F*_(2, 79.80)_ = 3.58, *p* < 0.05) with *post hoc* analyses highlighting lower amplitude in susceptible (−15.4%; *p* < 0.05; Figure 4A) and resilient (−38.7%; *p* < 0.001; Figure 4A) mice after CSDS, but not in control mice (*p* > 0.05; Figure 4A). Further analyses also suggest that resilient mice (*M* = −120.70, *SE* = 11.32) exhibit significantly lower a-wave amplitude compared to susceptible (*M* = −156.98, *SE* = 7.31; *p* < 0.01; Figure 5A) and control (*M* = −170.11, *SE* = 9.57; *p* < 0.01; Figure 5B) mice. The same effect was observed at 1.37 log cd.s/m^2^ (Figures 5A,B). Our analyses of the a-wave implicit time (time by phenotype interaction: *F*_(2,89.39)_ = 3.55, *p* < 0.05) also show that 10 days of social defeat significantly prolong the implicit time in resilient (+0.90 ms; *p* < 0.05; Figure 4B) mice, but induces no effect in susceptible (+0.14 ms; *p* > 0.05; Figure 4B) and control (−0.46 ms; *p* > 0.05; Figure 4B) mice. Accordingly, resilient mice showed a significantly longer a-wave implicit time (*M* = 20.70, *SE* = 0.30) than susceptible (*M* = 19.88, *SE* = 0.19; *p* < 0.05; Figure 5C) and control (*M* = 19.79, *SE* = 0.25; *p* < 0.05; Figure 5D) mice after CSDS. This phenomenon persisted at brighter luminance steps up to 2.86 log cd.s/m^2^ (Figures 5C,D). In contrast, analysis of the b-wave amplitude showed no time by phenotype interaction, but a significant main effect of time (*F*_(1,79.98)_ = 7.71, *p* < 0.01; Figure 4C) with a general pre-post decrease of 10.4% in all groups, therefore supporting an age effect. A similar lack of time by phenotype interaction was found for the b-wave implicit time (Figure 4D), however no main effect of time was established. Finally, analysis of the total OP amplitude (time by phenotype interaction: *F*_(2,81.51)_ = 3.84, *p* < 0.05) highlighted that CSDS induces a decrease of 43.4% in resilient (*p* < 0.01; Figure 4E) mice, but had no effect in neither the susceptible (−7.0%; *p* > 0.05; Figure 4E) nor control (+4.4%; *p* > 0.05; Figure 4E) groups. As a result, resilient mice exhibited a significantly lower total OP amplitude (*M* = 183.29, *SE* = 28.78) than susceptible (*M* = 281.20, *SE* = 18.41; *p* < 0.01; Figure 5E) and control (*M* = 315.33, *SE* = 23.95; *p* < 0.001 Figure 5F) mice after CSDS. This phenomenon was generalized throughout almost all the recorded luminance steps from 0.37–2.39 log cd.s/m^2^ (Figures 5E,F). To summarize, our analyses in scotopic condition suggest that male resilient mice exhibit lower and longer a-wave amplitudes and implicit times, respectively, along with lower total OP amplitudes compared to susceptible and control mice (refer to Supplementary Table 14).

**Figure 6:**
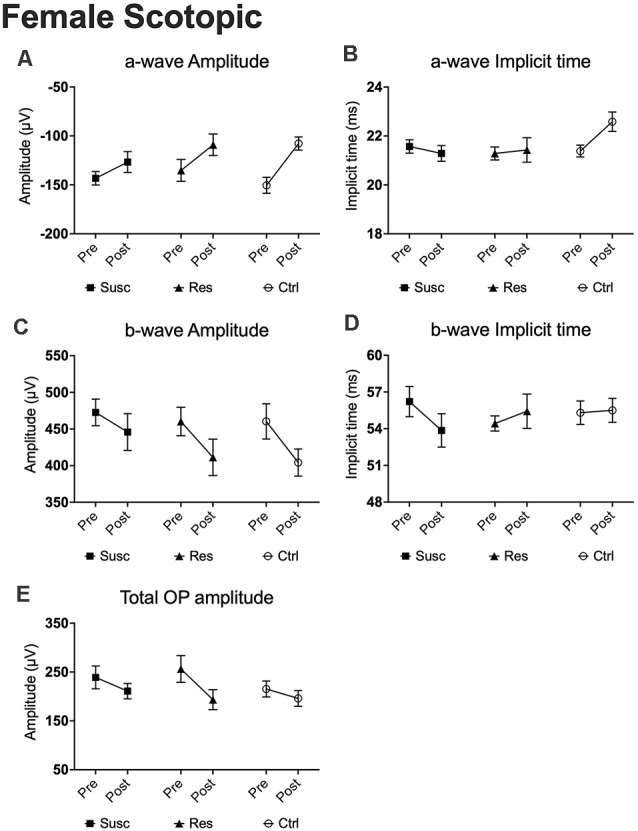
The evolution of each ERG component under the scotopic condition at baseline and after CSDS in female mice representing the susceptible, resilient, and control a-wave amplitude **(A)**, a-wave implicit time **(B)**, b-wave amplitude **(C)**, b-wave implicit time **(D)**, and total OP amplitude **(E)**. Significance was determined using linear mixed model analysis with LSD multiple comparisons test. Symbols and bars represent the group average ± SEM; *n*-values are given in the “Materials and Methods” section.

In females, analyses of the a-wave amplitude at 1.89 log cd.s/m^2^ revealed no significant time by phenotype interaction, but a significant main effect of time (*F*_(1,74.00)_ = 13.57, *p* < 0.001) with the three groups showing lower a-wave amplitude following CSDS (Figure 6A). Noticeably, this age effect was observed on a range of luminance from 1.37–2.86 log cd.s/m^2^. We found a similar lack of interaction for the a-wave implicit time (Figure 6B) with no main effect of time at 1.89 log cd.s/m^2^, although a significant interaction was identified at brighter luminance (2.39 log cd.s/m^2^; Supplementary Table 7). Regardless, our analyses of the b-wave amplitude highlighted a main effect of time (*F*_(1,73.35)_ = 5.95, *p* < 0.05; Figure 6C) that persisted at brighter luminance up to 2.86 log cd.s/m^2^ while no time by phenotype interaction nor the main effect of time were established for b-wave implicit time (Figure 6D). Finally, we identified a significant main effect of time (*F*_(1,60.30)_ = 4.95, *p* < 0.05) in the total OP amplitude displaying an overall decrease of 15.5% in all mice at post-stress, whether they were socially defeated or not (Figure 6E). In summary, our results in the female scotopic conditions point toward the main effect of age on a- and b-wave amplitudes and total OP amplitude with no effects of CSDS on rod activity.

### Photopic Condition (Cone ERG)

**Figure 7:**
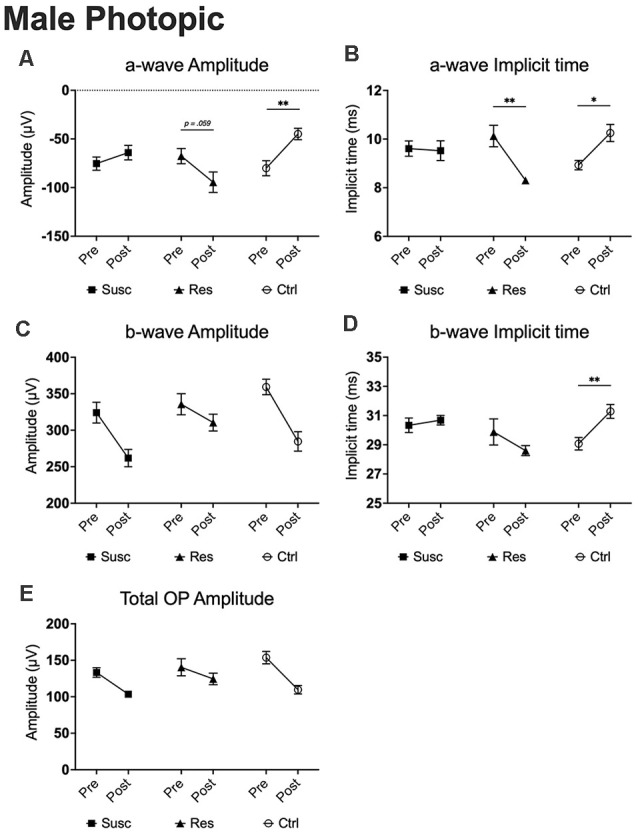
The evolution of each ERG component under the photopic condition at baseline and after CSDS in male mice representing the susceptible, resilient, and control a-wave amplitude **(A)**, a-wave implicit time **(B)**, b-wave amplitude **(C)**, b-wave implicit time **(D)**, and total OP amplitude **(E)**. Significance was determined using linear mixed model analysis with LSD multiple comparisons test. Symbols and bars represent the group average ± SEM; *p < 0.05, **p < 0.01; *n*-values are given in the “Materials and Methods” section.

In males, our analyses at 2.86 cd.s/m^2^ showed time by phenotype interactions for both a-wave amplitude (*F*_(2,78.80)_ = 5.79, *p* < 0.01) and implicit time (*F*_(2,79.59)_ = 6.93, *p* < 0.01). Interestingly, 10 days of CSDS induced a higher amplitude (−39.7%; *p* = 0.059; Figure 7A) and a shorter implicit time (−1.83 ms; *p* < 0.01; Figure 7B) in resilient mice while the opposite was observed in the non-defeated control mice demonstrating a lower amplitude (+43.9%; *p* < 0.01; Figure 7A) and a prolonged implicit time (+1.32 ms; *p* < 0.05; Figure 7A). As a result, the a-wave amplitude and implicit time in mice becoming resilient to CSDS (amplitude: *M* = −94.48, *SE* = 9.71; implicit time: *M* = 8.30, *SE* = 0.47) diverged significantly from those becoming susceptible (amplitude: *M* = −64.07, *SE* = 6.70; *p* < 0.05; implicit time: *M* = 9.52, *SE* = 0.32; *p* < 0.05) and the control (amplitude: *M* = −44.92, *SE* = 8.86; *p* < 0.001; implicit time: *M* = 10.25, *SE* = 0.43; *p* < 0.01) mice. In contrast, analyses (2.86 cd.s/m^2^) of the b-wave amplitude and total OP amplitude revealed no time by phenotype interaction, but significant main effects of time (b-wave amplitude: *F*_(1,76.69)_ = 21.53, *p* < 0.001; Figure 7C; total OP amplitude: *F*_(1,70.18)_ = 24.20, *p* < 0.001; Figure 7E) with a general pre-post decrease of 15.9% (*p* < 0.001) and 21.0% (*p* < 0.001), respectively. This age effect persisted for every luminance step (0.88–2.86 log cd.s/m^2^) in both b-wave and total OP amplitudes. Finally, our analyses (2.86 cd.s/m^2^; time by phenotype interaction: *F*_(2,74.47)_ = 4.64, *p* < 0.05) also show that, in control mice, the passage of time (10 days) significantly prolongs the b-wave implicit time (+2.22 ms; *p* < 0.01; Figure 7D) while the 10 days of social defeat induced no effect in neither susceptible (+0.35 ms; *p* > 0.05; Figure 7D) nor resilient (−1.28 ms; *p* > 0.05; Figure 7D) mice. As a result, resilient mice showed not only a significantly shorter b-wave implicit time (*M* = 20.70, *SE* = 0.30) than control (*M* = 19.79, *SE* = 0.25; *p* < 0.05; Figure 5H) mice after CSDS, but also than susceptible (*M* = 19.88, *SE* = 0.19; *p* < 0.05; Figure 5G) mice. This phenomenon persisted in all recorded luminance steps (0.88–2.86 log cd.s/m^2^; Figures 5G,H). To summarize the findings in male photopic ERGs, resilient mice persistently exhibited shorter b-wave implicit time compared to controls and susceptibles, and age effects were observed in both b-wave and total OP amplitudes (refer to Supplementary Table 14).

In contrast, the analyses of the photopic condition in females revealed no significant time by phenotype interaction nor the main effect of time for all of ERG components (all *p* > 0.05; Supplementary Tables 9–13), suggesting that 10 days of social defeat has no functional impact on the cone activity in females, whether they were socially defeated or not.

## Predictive Ability of the Electroretinography

**Figure 8:**
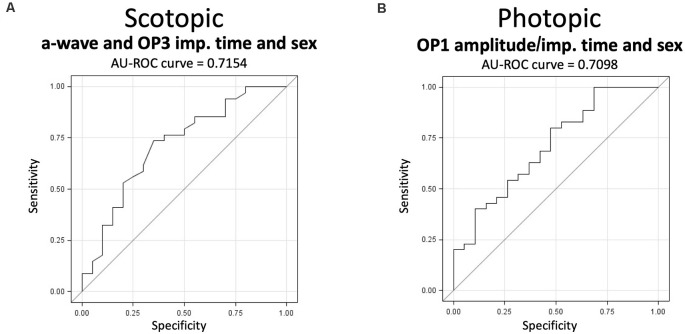
The capacity of the scotopic and photopic ERGs to predict the expression of stress susceptibility and resilience from baseline signals. **(A)** The scotopic model fitting the scotopic a-wave and third OP implicit time with sex has an AU-ROC curve of 0.7154 (*p* < 0.05). **(B)** The photopic model fitting the first photopic OP amplitude and implicit time with sex has an AU-ROC curve of 0.7098 (*p* < 0.05). Statistical significance was determined using multiple backward logistic regressions and was set at *p* < 0.05; *n*-values are given in the “Materials and Methods” section.

Finally, we tested our capacity to predict the expression of stress susceptibility and resilience based on baseline ERG signals using receiver operating characteristic curves combined with multiple backward logistic regressions. Performing the analyses on males and females separately failed to provide predictive models due to a general lack of power (data not shown). For this reason, we performed the same analyses combining both sexes to maximize the statistical power. These analyses highlighted a significant fitted model for both the scotopic (*p* > 0.05) and photopic (*p* > 0.05) ERGs individually. In scotopic, the backward procedure isolated a model fitting the a-wave implicit time and the third OP (OP3) implicit time using sex as a covariate. The AU-ROC curve of this model reached 0.7154 (Figure 8A) with a sensitivity and specificity of 85.3 and 30.0, respectively. In photopic, our analysis identified a model fitting the amplitude and implicit time of the first OP (OP1) of the ERG signals using sex as a covariate (Figure 8B). This model has an estimated AU-ROC curve of 0.7098 with a sensitivity and specificity of 82.9 and 31.6, respectively. Overall, results from these analyses suggest that the ERG can predict the expression of susceptibility or resilience phenotypes in male or female mice subjected to CSDS.

## Discussion

In this study, we tested if the phenotypes of susceptibility and resilience to CSDS are associated with specific ERG alterations and whether baseline ERG signals could detect which males and females undergoing CSDS would develop such phenotypes. Our results show that CSDS induces changes in the activity of the retina in males but not females. Additionally, our analysis suggests that baseline ERG measures could be used to predict male and female phenotype after 10 days of social stress.

The retina is a highly heterogeneous tissue composed of different cell types, each playing a specific role in the integration of visual information. Consequently, interfering with the coordinated activity of these cells might change the processing of visual information and ultimately, alters the perception of the environment. Accordingly, our results showed that stress resilience in males associates with multiple ERG alterations in rods a-waves and OPs as well as in cones b-waves. Interestingly, all the a-wave alterations exhibited by the resilient mice were within a range of stimuli approaching rod saturation (≥1.37 log of luminance). As the a-wave measured at brighter luminance is widely associated with the activity of the photoreceptors (Penn and Hagins, 1969; Hood and Birch, 1993), we could hypothesize that the depressed and prolonged a-waves observed in resilient mice might be indicative of a reduced and delayed rods’ hyperpolarization in response to light stimulus. This variation in time may also result from a delayed intrusion of the b-wave (Murakami and Kaneko, 1966; Jamison et al., 2001) likely reflecting a slower depolarization of ON-bipolar cells following the decrease in glutamate release from the rods (Sieving et al., 1994). Additionally, the changes in rods OP amplitudes observed in males resilient to CSDS might be reflective of a greater interaction between the amacrine and ganglion cells within the retina (Wachtmeister, 1998), while the resilience-associated changes in cones b-wave implicit times presumably imply that the ON-bipolar cellular activity accelerates following glutamate release in the cones as a coping mechanism to social stress (Miller and Dowling, 1970; Sieving et al., 1994). As a- and b-waves are complex signals integrating the activity of several cell types, future studies should consider assessing the underlying changes in the cell types associated with resilience. Together, our results support the concept that resilience to social stress results from active and adaptative functional and molecular mechanisms allowing mice to cope with and develop adaptative behavioral responses to stress (Krishnan et al., 2007; Dias et al., 2014). In our study, this associates with a series of functional changes in the sensory processing and, presumably, the perception of environmental threats. On the other hand, the lack of effect in susceptible mice is surprising given that susceptibility to social stress has been shown to involves sex-specific molecular alterations in several pathways regulating stress responses (Krishnan et al., 2007; Iniguez et al., 2014, 2018). Overall, our findings suggest that social stress induces a wide range of adaptative responses (Blanchard et al., 2001; Viana Borges et al., 2019), not only in the brain but also in peripheral organs, and support the use of the ERG to detect these alterations in the retina.

Dopamine (DA) and serotonin (5-HT) are central players in the neurochemical imbalance hypothesis underlying the pathophysiology of depression (Belmaker and Agam, 2008; Saini et al., 2008) in humans and stress responses in susceptible and resilient mice to CSDS (Keeney et al., 2006; Watt et al., 2009; Tse et al., 2014; Iniguez et al., 2016; Favoretto et al., 2020). Previous studies using transgenic mice showed that alterations in central monoamines impact retinal activity in both humans and animal models (Fornaro et al., 2011; Lavoie et al., 2014b). That being said, the mechanisms linking alterations of central DA and 5-HT circuits with the activity of the retina are still unclear. Interestingly, evidence of direct histaminergic and serotonergic projections from the brain to the retina (referred as retinopetal neurons) have previously been reported in mammals (Gastinger et al., 2006a,b; Ortiz et al., 2017). In mice, retinal dopaminergic cells-mostly the amacrine cells (Reis et al., 2007)—are among the targets of the histaminergic retinopetal axons (Greferath et al., 2009; Frazao et al., 2011). Since light adaptation in the retina is modulated by DA (Witkovsky, 2004) and that changes in central DA can affect histamine release (Horner et al., 2007; Yanovsky et al., 2011), the ERG alterations associated with stress resilience in our study might be accounted, in part, by the influence of the histaminergic retinopetal axons. The 5-HT also acts as a neuromodulator in the retina (Vaney, 1986) and its activity could potentially be modulated by serotonergic retinopetal neurons emerging from the dorsal raphe nucleus (DRN; Lima and Urbina, 1998; Gastinger et al., 2006b; Lorincz et al., 2008). In fact, not only bilateral administration of the neurotoxic agent 5-7-dihydroxytryptamine (5,7-DHT) into the DRN has been associated with lower 5-HT and serotonin transporter (SERT) contents in the retina (Lima and Urbina, 1998), electrophysiological stimulation of the DRN in rats have also been shown to change retinal activity (Lorincz et al., 2008). It is therefore legitimate to hypothesize a potential relationship between the retinopetal projections and the ERG alterations induced by CSDS. Future studies should consider the role of these projections to better understand how changes affecting the central nervous system impact the retina in the context of chronic stress.

Our findings highlighted the existence of sexual dimorphism in the activity of the retina under basal conditions and following social stress, which is in contrast with previous studies in C57BL/6 mice (Mazzoni et al., 2019). This divergence may be explained by experimental considerations such as differences in the sample size and the statistical approaches used in both studies. Nevertheless, considering that many sex-specific features were identified in gene expression throughout mouse’s lifespan (Du et al., 2017) and that plasticity of the visual system still occurs during adulthood in mice (Antonini et al., 1999; Keck et al., 2008), it should not be inconceivable to find sex differences in basal ERG responses. Another possibility explaining male and female ERG differences relate to variations in hormonal status (Chaychi et al., 2015) although no differences in female estrous cycles were found in our study, reducing the impact of this potential factor. The lack of effect in females specifically is surprising as a growing body of evidence describes distinct physiological (Rincon-Cortes et al., 2019; Zuloaga et al., 2020), functional (Hastings et al., 2004), and molecular (Labonte et al., 2017) alterations induced by chronic stress in males and females. It is also believed that these differences may be part of the reasons underlying why males and females respond differently to stress (Kornstein et al., 2000; Khan et al., 2005). It will be important in future studies to further investigate these differences using, for example, molecular approaches, pupillometry, or other waveform components such as the photopic negative response (PhNR) or the c-wave.

Past and recent clinical and preclinical studies highlighted the fact that no reliable biomarkers or specific endophenotypes exist for MDD and associated diseases (Belmaker and Agam, 2008; Saini et al., 2008; Miller et al., 2009; Otte et al., 2016; Menke, 2019). A similar lack of predictive capabilities exists in preclinical research using animal models. Here, we provided results suggesting that the ERG might predict mice’s phenotype to social stress with an efficiency of roughly 71.5% when using a combination of ERG components under both scotopic and photopic conditions separately. While these results still need to be replicated, this efficiency remains promising for a first attempt in predicting, ahead of stress, the expression of susceptibility and resilience using the ERG. This is indeed in line with previous results using ERG from which SZ patients were differentiated from individuals suffering from bipolar disorder with 86% accuracy (Hebert et al., 2020). Importantly, our results, along with those in clinical research using ERG (Lam et al., 1992; Fornaro et al., 2011; Fam et al., 2013; Hebert et al., 2017), suggest that retinal activity might represent an endophenotype which could be used to determine whether mice or humans facing social stress are more likely to develop specific aspects of the disease. This is of the utmost importance as by combining the predictive capabilities of ERG with molecular or functional studies, one could specifically target some of the systems directly involved in the development of stress resilience or susceptibility and perform invasive tests (e.g., collecting brain tissue) in unstressed animals or while they are developing these phenotypes. That being said, although we pooled males and females for the predictive analyses, our statistical model was still limited by our sample size which did not allow us to reach sufficient power to establish robust predictions, therefore suggesting that the use of a larger sample size would be beneficial in future studies.

One surprising result in our study is that prolonged cone b-wave implicit times were observed only in control mice-as if it was a typical effect of age-with no effect in stressed mice, therefore, suggesting that CSDS hampers this effect. Interestingly, prolonged cone b-wave implicit times appear to be the most reported manifestation in psychiatric diseases-including MDD-in the human retina (Fountoulakis et al., 2005; Hebert et al., 2010, 2017, 2020), thus being in contrast with our results. It is difficult to explain this difference, but it should first be noticed that findings in the retinal anomalies occurring during MDD in humans are still inconsistent. For instance, in a clinical study investigating MDD and SZ, no alterations were reported when comparing MDD patients to healthy controls (Demmin et al., 2020). It should also be noticed that, in contrast with diurnal humans, mice are nocturnal mammals with different spectral sensitivity inherent to the distinct population and density of photoreceptors in their eyes (Peirson et al., 2018). These distinctions might explain some of the differences observed in our studies and previous ERG analyses performed in clinical human populations (Fornaro et al., 2011; Hebert et al., 2017). This consideration may become even more important considering that most significant results in humans originate from photopic (light-adapted) ERGs (Fornaro et al., 2011; Hebert et al., 2017) while most of our results were obtained in the scotopic (dark-adapted) condition. These facts should be considered when interpreting the translational value of our findings.

Overall, the search for reliable biomarkers and endophenotypes of stress responses has been shaping clinical and preclinical research for the last decades with still limited success (Belmaker and Agam, 2008; Saini et al., 2008; Miller et al., 2009; Otte et al., 2016; Menke, 2019). Here, we present results suggesting that not only social stress brings forth changes in retinal activity that associate with the expression of resilience in a mouse model aiming to induce depressive-like behaviors, but also that the ERG can be used to predict the mouse phenotype to social stress. The CSDS is, however, one of many mouse models (Goodwill et al., 2019; Scarpa et al., 2020) and previous molecular studies highlighted the capacity of different mouse models to reproduce not only the clinical manifestations but also the molecular changes relevant to MDD in males and females (Labonte et al., 2017; Scarpa et al., 2020). Thus, in upcoming studies, it will be important to determine whether different types of stress induce similar ERG alterations and whether this non-invasive tool can be used to predict the expression of different behavioral alterations triggered by the distinct behavioral paradigms used to model depression in males and females. Further enhancing the predictive ability of the ERG would also allow monitoring the development of the pathology during the progression of stress in mice. Ultimately, the exciting options offered by the ERG in preclinical research paves the way to novel research avenues for studying the molecular and functional mechanisms underlying the expression of stress responses and develop new therapeutic options to treat MDD more efficiently.

## Data Availability Statement

The raw data supporting the conclusions of this article will be made available by the authors without undue reservation.

## Ethics Statement

This animal study was reviewed and approved by the Université Laval’s Institutional Animal Care Committee in respect with the Canadian Council on Animal Care guidelines. This study was carried out in compliance of the Animal Research: Reporting of *in vivo* Experiments (ARRIVE) guidelines. Additionally, all efforts were made in this study to reduce the number of animals used and to minimize their suffering.

## Author Contributions

BL, MH, and EA conceived and designed this research. EA and A-AL performed the experiments and ran the behavioral testing. EA, SM, A-MG, and KF carried out the analyses and prepared figures. A-AL, TB, FQ, KA, AB, and EA performed tissue dissections. EA and BL wrote the manuscript. All authors contributed to the article and approved the submitted version.

## Conflict of Interest

The authors declare that the research was conducted in the absence of any commercial or financial relationships that could be construed as a potential conflict of interest.

## Publisher’s Note

All claims expressed in this article are solely those of the authors and do not necessarily represent those of their affiliated organizations, or those of the publisher, the editors and the reviewers. Any product that may be evaluated in this article, or claim that may be made by its manufacturer, is not guaranteed or endorsed by the publisher.
