# Supplementary material for: Sex-Specific Retinal Anomalies Induced by Chronic Social Defeat Stress in Mice
Source: Front Behav Neurosci. 2021 Aug 12;15:714810. doi: 10.3389/fnbeh.2021.714810 (PMC8415161; doi:10.3389/fnbeh.2021.714810)
Supplement: Supplementary file 1 [file Data_Sheet_1.pdf]

Sex-specific Retinal Anomalies Induced by Chronic Social Stress in Mice: Supplementary Material

Detailed ERG analyses

For both scotopic and photopic recordings, average signals were calculated from all ERG traces collected for each flash stimulation in each mouse using Espion 3.0.1 (Diagnosys LLC, Lowell, MA). To remove OP contamination from the a- and b-wave measurements, low-frequency waves were isolated from the scotopic signals (**Figure 1c**) of each mouse applying a band-stop filter from 75 to 300 Hz using the Butterworth method from the Infinite Impulse Response (IIR) filter tool in OriginPro 2020 (OriginLab Corp., Northampton, MA). Such filtering is promoted by the International society for clinical electrophysiology of vision (McCulloch et al., 2015) and was previously used in mice by Lavoie, et al. (Lavoie et al., 2014). Conversely, OPs were extracted from both scotopic and photopic ERGs (**Figure 1 d, e**) of each mouse by removing the low frequencies applying a digital band-pass filtering from 75 to 300 Hz (Lavoie et al., 2014; McCulloch et al., 2015) using the Fast Fourier Transform (FFT) tool from the same software. Amplitudes and implicit times of the scotopic and photopic OPs and scotopic a- and b-waves were measured for each resultant wave using OriginPro 2020 (OriginLab Corp., Northampton, MA). The photopic a- and b-waves were measured from the non-filtered signals using the same software. Note that mice were identified by identifier (ID) numbers during all ERG recordings as well as during the signal processing and assessment in order to generate unbiased data. Outliers for each group were then identified using the GraphPad's QuickCalcs Grubb's test (GraphPad Software Inc., San Diego, CA) and removed when applicable. Statistical analyses were performed for every luminance step on the total OP amplitude (summation of the peak amplitude measured in the first four OPs and first two OPs in scotopic and photopic conditions, respectively) and the a- and b-wave amplitudes and implicit times with SPSS Statistics 27.0 (IBM Corp., Armonk, NY). Scotopic and photopic ERGs were analyzed separately.

The ERG data was presented as the mean and standard error of each parameter. For both scotopic and photopic ERGs, a linear mixed model analysis was performed to compare male and female susceptible, resilient, and control mice at baseline using the recorded luminance step (scotopic: -0.020 to 2.859 log cd.s/m^2; photopic: 0.885 to 2.859 log cd.s/m^2) as a covariate. Post hoc were analyzed using Fisher's least significant difference (LSD) multiple comparisons test. Variations in ERG parameters for each sex were then tested separately using linear mixed model analyses in which phenotype (susceptible, resilient, and control) and time of assessment (pre-stress versus post-stress) were included as main factors. Mean and standard error of each parameter were presented for each distinct level combination of the factors. The LSD multiple comparisons test was used in post hoc. All p-values under .05 were considered as significant.

Lavoie, J., Illiano, P., Sotnikova, T. D., Gainetdinov, R. R., Beaulieu, J. M., and Hebert, M. (2014). The electroretinogram as a biomarker of central dopamine and serotonin: potential relevance to psychiatric disorders. *Biol. Psychiatry* 75, 479-486. doi: 10.1016/j.biopsych.2012.11.024

McCulloch, D. L., Marmor, M. F., Brigell, M. G., Hamilton, R., Holder, G. E., Tzekov, R., and Bach, M. (2015). Erratum to: ISCEV Standard for full-field clinical electroretinography (2015 update). *Doc. Ophthalmol.* 131, 81-83. doi: 10.1007/s10633-015-9504-z

Supplemental table 1 | Distribution of the social interaction ratios upon the estrous cycle of the susceptible, resilient, and control mice

	Susceptible		Resilient		Control		Total	
	n	*M*	*n*	*M*	*n*	*M*	*n*	*M*
**Reproductive**								
Pro-estrus	3	.586	4	1.64	1	1.64	8	1.35
Estrus	5	.579	6	1.33	7	1.07	18	1.02
Total	8	.581	10	1.46	8	1.11	26	1.08
**Non-reproductive**								
Metestrus	4	.720	4	1.32	4	1.41	12	1.12
Diestrus	2	.699	0	-	1	1.89	3	.998
Total	6	.707	4	1.32	5	1.51	15	1.14

Supplemental Table 1. Distribution of the SI ratios upon the estrous cycle of the susceptible, resilient, and control female mice. The SI ratio is calculated from the time spent in the interaction zone (time in interaction zone with social target CD-1 / time in interaction zone without the social target) during the social interaction test. *M* = mean.

Supplemental table 2 | Two-way mixed ANOVA results of the scotopic condition at -0.02 cd.s/m^2 of luminance

Sex	Wave	Parameter	Time	Susceptible (M = 25, F = 14) *M (SE)*	Resilient (M = 10, F = 14) *M (SE)*	Control (M = 14, F = 13) *M (SE)*	Fixed effet: Phenotype *P (F)*	Fixed effet: Time *P (F)*	Fixed effet: Phenotype*Time *P (F)*
**Male (*n* = 49)**	a-wave	Amplitude	Pre	-17.73 (1.89)	-16.00 (2.51)	-19.50 (2.67)	*.6298 (0.47)*	*.7068 (0.14)*	*.9938 (0.01)*
			Post	-16.84 (2.76)	-15.41 (4.46)	-18.20 (3.15)			
		Implicit time	Pre	29.12 (0.65)	29.78 (0.89)	31.25 (0.95)	*.1745 (1.80)*	*.5913 (0.29)*	***.0206 (4.16)***
			Post	29.20 (0.66)	31.80 (1.14)	28.00 (0.81)			
	b-wave	Amplitude	Pre	248.17 (15.85)	259.81 (21.13)	281.27 (22.41)	*.3538 (1.06)*	*.2847 (1.17)*	*.9083 (0.10)*
			Post	236.78 (14.26)	243.33 (29.68)	254.56 (16.26)			
		Implicit time	Pre	72.35 (1.12)	70.11 (1.54)	69.20 (1.47)	*.1246 (2.16)*	*.3349 (0.95)*	*.9105 (0.09)*
			Post	70.35 (1.22)	69.50 (2.52)	67.90 (1.59)			
	Total OP	Amplitude	Pre	107.23 (9.83)	112.94 (13.46)	198.27 (26.92)	*.0057 (6.00)*	*.0013 (12.41)*	***.0266 (4.03)***
			Post	101.65 (6.34)	82.94 (13.08)	108.26 (7.55)			
**Female (*n* = 41)**	a-wave	Amplitude	Pre	-16.45 (3.12)	-10.47 (3.37)	-9.29 (2.92)	*.4966 (0.83)*	*.6328 (0.27)*	*.7061 (0.38)*
			Post	-12.70 (5.52)	-3.90 (9.57)	-12.38 (6.76)			
		Implicit time	Pre	32.57 (1.38)	32.00 (1.49)	31.25 (1.29)	*.5476 (0.71)*	*.5555 (0.42)*	*.4451 (1.00)*
			Post	34.33 (2.80)	29.00 (4.85)	37.00 (3.43)			
	b-wave	Amplitude	Pre	226.63 (25.29)	206.71 (27.31)	204.20 (27.31)	*.9844 (0.02)*	*.7281 (0.14)*	*.8629 (0.15)*
			Post	192.67 (39.33)	197.22 (68.12)	209.55 (48.17)			
		Implicit time	Pre	78.57 (1.75)	75.00 (1.75)	75.17 (1.89)	*.7518 (0.30)*	*.7996 (0.07)*	*.4905 (0.84)*
			Post	75.25 (4.79)	74.00 (9.58)	83.00 (6.77)			
	Total OP	Amplitude	Pre	101.98 (12.57)	79.23 (11.25)	65.42 (11.25)	*.9192 (0.08)*	*.4673 (0.61)*	***.0478 (6.46)***
			Post	58.85 (13.95)	55.46 (14.06)	88.27 (13.95)			

Supplemental Tables 2-8. Summary of the two-way mixed model ANOVA results from all recorded densities of light for the scotopic condition comparing means and standard errors from susceptible, resilient, and control mice. The tables also display the fixed effect of each interaction from the analysis. *M* = mean; *SE* = standard error, *P* = p-value, *F* = Fisher's statistic. Significance is set at .05.

Supplemental table 3 |Two-way mixed ANOVA results of the scotopic condition at 0.37 cd.s/ m² of luminance

				Susceptible (M = 25, F = 14)	Resilient (M = 10, F = 14)	Control (M=14,F=13)	Fixed effet		
							Phenotype	nme	*Phenotype"Time*
**Sex**	**Wave**	**Parameter**	**Time**	*M (SE)*	*M (SE)*	*M (SE)*	*p (F)*	*p (F)*	*p (F)*
**Male (n = 49)**	a-wave	Amplitude	Pre	-29.19 (2.44)	-25.61 (3.95)	-23.18 (2.99)	*.6698 (0.40)*	*.1006 (2.17)*	***.0119 (4.71)***
			Post	-17.73 (2.22)	-20.30 (3.24)	-27.79 (2.76)			
		Implicit time	Pre	27.91 (0.42)	29.44 (0.66)	26.57 (0.53)	*.1888 (1.11)*	*.2577 (1.30)*	***.0269 (3.80)***
			Post	27.00 (0.53)	27.33 (0.75)	27.91 (0.67)			
	b-wave	Amplitude	Pre	299.00 (14.88)	309.81 (24.11)	325.25 (18.22)	*.1926 (1.68)*	*.1343 (2.29)*	*.6714 (0.40)*
			Post	286.55 (14.20)	266.24 (19.52)	313.06 (17.66)			
		Implicit time	Pre	67.82 (1.11)	64.78 (1.74)	66.07 (1.39)	*.4658 (0.77)*	*.6151 (0.25)*	*.1985 (1.65)*
			Post	65.44 (1.17)	67.44 (1.66)	64.00 (1.44)			
	Total OP	Amplitude	Pre	163.70 (12.89)	191.10 (20.39)	161.67 (17.39)	*.3342 (1.11)*	*.0254 (5.21)*	***.0106 (4.85)***
			Post	153.99 (10.08)	92.80 (22.54)	177.25 (12.05)			
**Female (n = 41)**	a-wave	Amplitude	Pre	-20.46 (2.33)	-19.66 (2.57)	-21.31 (2.23)	*.6187 (0.49)*	*.2152 (1.58)*	*.6054 (0.51)*
			Post	-20.56 (2.98)	-16.11 (3.30)	-15.59 (4.04)			
		Implicit time	Pre	28.92 (0.72)	30.67 (0.83)	29.38 (0.69)	*.3155 (1.18)*	*.6414 (0.22)*	*.6420 (0.45)*
			Post	29.36 (0.85)	30.10 (0.89)	30.50 (1.15)			
	b-wave	Amplitude	Pre	263.62 (16.23)	262.23 (17.94)	266.14 (17.02)	*.8538 (0.16)*	*.1719 (1.93)*	*.8128 (0.21)*
			Post	253.36 (20.83)	238.46 (23.03)	228.85 (28.21)			
		Implicit time	Pre	71.83 (1.08)	72.00 (1.19)	70.18 (1.13)	*.7030 (0.36)*	*.6826 (0.17)*	*.1981 (1.69)*
			Post	70.00 (1.91)	71.09 (1.91)	74.67 (2.59)			
	Total OP	Amplitude	Pre	130.54 (13.81)	134.10 (13.02)	115.73 (12.35)	*.9750 (0.03)*	*.5262 (0.42)*	*.5265 (0.66)*
			Post	112.46 (15.26)	105.63 (19.30)	133.08 (30.51)			

Supplemental table 4 | Two-way mixed ANOVA results of the scotopic condition at 0.88 cd.s/m^2 of luminance

				Susceptible (M = 2S, F = 14)	Resilient (M = 10, F = 14)	Control (M = 14, F = 13)	Fixed effet		
							Phenotype	*Time*	*Phenotype *Time*
**Sex**	**Wave**	**Parameter**	**Time**	*M (SE)*	*M (SE)*	*M (SE)*	*p (F)*	*p (F)*	*p (F)*
**Male (*n* =49)**	a-wave	Amplitude	Pre	-57.41 (4.76)	-63.35 (7.93)	-59.53 (6.36)	*.2841 (1.28)*	*.0003 (14.37)*	*.1181 (2.20)*
			Post	-43.43 (3.15)	-32.44 (4.98)	-53.80 (4.20)			
		Implicit time	Pre	26.40 (0.24)	26.00 (0.40)	24.86 (0.32)	*.0431 (3.26)*	*.1396 (2.22)*	***.0202 (4.07)***
			Post	25.44 (0.26)	25.10(0.41)	25.50 (0.34)			
	6-wave	Amplitude	Pre	359.24 (15.46)	386.61 (25.77)	417.68 (20.67)	***.0420 (3.30)***	***.0394 (4.38)***	*.1684 (1.82)*
			Post	363.08 (11.30)	326.82 (17.87)	379.85 (15.11)			
		Implicit time	Pre	62.40 (0.98)	60.67 (1.63)	61.57 (1.31)	*.5685 (0.57)*	*.2573 (1.30)*	*.8182 (0.20)*
			Post	60.88 (0.94)	60.50 (1.49)	59.64 (1.26)			
	Total OP	Amplitude	Pre	233.44 (17.20)	261.49 (26.89)	262.10 (26.89)	*.0978 (2.41)*	*.0067 (7.82)*	***.0344 (3.54)***
			Post	220.55 (12.65)	146.12 (20.66)	247.06 (17.19)			
**Female (*n* = 41)**	a-wave	Amplitude	Pre	-39.58 (4.20)	-36.10 (4.20)	-39.39 (4.36)	*.7119 (0.34)*	*.2126 (1.58)*	*.7863 (0.24)*
			Post	-36.66 (3.63)	-33.96 (3.78)	-31.81 (4.37)			
		Implicit time	Pre	26.93 (0.53)	27.64 (0.53)	27.31 (0.55)	*.7096 (0.34)*	*.2144 (1.57)*	*.9046 (0.10)*
			Post	27.79 (0.57)	28.00 (0.61)	27.89 (0.71)			
	6-wave	Amplitude	Pre	335.63 (16.60)	328.88 (16.60)	332.55 (17.93)	*.4264 (0.86)*	*.0795 (3.18)*	*.4772 (0.75)*
			Post	324.54 (18.99)	312.17 (20.52)	276.46 (23.69)			
		Implicit time	Pre	67.29 (1.36)	63.64 (1.36)	66.42 (1.47)	*.5049 (0.69)*	*.7675 (0.09)*	*.2908 (1.26)*
			Post	64.50 **(1.51)**	65.50 (1.63)	66.22 (1.88)			
	Total OP	Amplitude	Pre	174.28 (18.42)	183.82 (17.56)	172.35 (17.56)	*.6971 (0.36)*	*.1528 (2.10)*	*.3081 (1.20)*
			Post	162.33 (15.38)	132.15 (16.13)	172.96 (20.82)			

Supplemental table 5 | Two-way mixed ANOVA results of the scotopic condition at 1.37 cd.s/m^2 of luminance

Sex	Wave	Parameter	Time	Susceptible (M = 25, F = 14)	Resilient (M = 10, F = 14)	Control (M=14,F=13)	Fixed effet		
							Phenotype	*Time*	*Phenotype *Time*
				M (SE)	*M (SE)*	*M (SE)*	*p (F)*	*p (F)*	*p (F)*
**Male (n = 49)**	a-wave	Amplitude	Pre	-110.99 (7.84)	-120.90 (12.40)	-108.43 (10.88)	*.2954 (1.24)*	*.0016 (10.64)*	***.0233 (3.94)***
			Post	-91.28 (5.58)	-66.18 (8.82)	-110.08 (7.45)			
		Implicit time	Pre	24.00 (0.24)	23.40 (0.39)	22.69 (0.34)	*.0832 (2.56)*	*.7819 (0.08)*	***.0220 (3.99)***
			Post	23.24 (0.20)	23.70 (0.32)	23.36 (0.27)			
	b-wave	Amplitude	Pre	432.79 (16.67)	440.09 (26.36)	489.86 (23.12)	*.0662 (2.80)*	***.0140 (6.30)***	.5667 *(0.57)*
			Post	412.01 (12.89)	395.80 (20.38)	432.29 (17.22)			
		Implicit time	Pre	57.32 (0.86)	57.00 (1.36)	56.77 (1.19)	.3505 *(1.06)*	*.3724 (0.80)*	*.3520 (1.06)*
			Post	56.92 (0.75)	54.30 (1.19)	57.50 (1.01)			
	Total OP	Amplitude	Pre	279.58 (17.19)	284.05 (26.63)	288.76 (25.39)	.0584 *(2.95)*	.0795 *(3.16)*	**.0604 *(2.91)***
			Post	277.62 (13.83)	191.70 (21.13)	292.91 (16.94)			
**Female (n = 41)**	a-wave	Amplitude	Pre	-79.83 (5.99)	-80.13 (5.99)	-82.09 (6.21)	.7306 *(0.32)*	***.0080 (7.44)***	*.5215 (0.66)*
			Post	-72.30 (6.43)	-66.99 (6.67)	-59.75 (7.25)			
		Implicit time	Pre	24.71 (0.42)	24.93 (0.42)	24.54 (0.43)	.7564 *(0.28)*	*.2930 (1.12)*	*.4120 (0.90)*
			Post	24.79 (0.45)	24.92 (0.46)	25.64 (0.50)			
	b-wave	Amplitude	Pre	406.17 (16.63)	404.53 (16.63)	401.90 (17.26)	*.6461 (0.44)*	***.0291 (4.97)***	*.7716 (0.26)*
			Post	385.27 (20.51)	368.40 (21.28)	352.92 (23.14)			
		Implicit time	Pre	60.29 (1.24)	59.93 (1.24)	60.08 (1.29)	.7866 *(0.24)*	*.8415 (0.04)*	*.6743 (0.40)*
			Post	58.86 (1.18)	60.00 (1.23)	60.82 (1.34)			
	Total OP	Amplitude	Pre	230.14 (17.63)	243.37 (17.63)	207.56 (16.88)	*.3625 (1.03)*	***.0104 (7.00)***	*.3048 (1.21)*
			Post	210.72 (18.68)	172.62(17.18)	180.12 (20.65)			

Supplemental table 6 | Two-way mixed ANOVA results of the scotopic condition at 1.89 cd.s/m^2 of luminance

				Susceptible (M=2S,F=14)	Resilient (M=10, F = 14)	Control (M=14,F=13)	Fixed effet		
							Phenotype	*Time*	*Phenotype"Time*
**Sex**	**Wave**	**Parameter**	**Time**	*M (SE)*	*M (SE)*	*M (SE)*	*p (F)*	*p (F)*	*p (F)*
**Male (n = 49)**	a-wave	Amplitude	Pre	-185.66 (10.63)	-196.76 (16.46)	-176.94 (13.91)	*.4830 (0.73)*	*.0003 (14.63)*	***.0325 (3.58)***
			Post	-156.98 (7.31)	-120.69 (11.32)	-170.11 (9.57)			
		Implicit time	Pre	20.33 (0.21)	19.80 (0.32)	19.64 (0.27)	*.1261 (2.12)*	*.3600 (0.85)*	***.0330 (3.54)***
			Post	19.88 (0.19)	20.70 (0.29)	19.79 (0.25)			
	b-wave	Amplitude	Pre	493.04 (21.07)	498.41 (32.64)	559.34 (27.59)	***.0309 (3.63)***	***.0068 (7.11)***	*.6487 (0.44)*
			Post	461.96 (14.55)	433.52 (22.55)	494.49 (19.05)			
		Implicit time	Pre	53.46 (0.52)	53.10 (0.80)	53.29 (0.68)	*.1717 (1.80)*	*.3458 (0.90)*	*.2743 (1.32)*
			Post	53.25 (0.87)	50.50 (1.34)	53.93(1.13)			
	Total OP	Amplitude	Pre	302.40 (21.00)	324.03 (33.57)	302.11 (27.93)	*.1604 (1.87)*	*.0225 (5.41)*	***.0255 (3.84)***
			Post	281.20 (18.41)	183.28 (28.78)	315.33 (23.95)			
**Female (n = 41)**	a-wave	Amplitude	Pre	-143.27 (8.86)	-135.24 (8.86)	-150.47 (9.19)	*.3880 (0.96)*	***.0004 (13.57)***	*.3924 (0.95)*
			Post	-126.70 (9.68)	-109.05 (9.68)	-107.76 (10.46)			
		Implicit time	Pre	21.57 (0.26)	21.29 (0.26)	21.38 (0.27)	*.1632 (1.87)*	*.2197 (1.54)*	*.1060 (2.33)*
			Post	21.29 (0.41)	21.43 (0.41)	22.58 (0.44)			
	b-wave	Amplitude	Pre	472.61 (20.26)	460.22 (20.26)	460.36 (21 02)	*.4141 (0.89)*	***.0172 (5.95)***	*.7862 (0.24)*
			Post	445.82 (22.91)	411.30 (22.91)	404.18 (24.75)			
		Implicit time	Pre	56.21 (0.96)	54.43 (0.96)	55.31 (1.00)	*.9128 (0.09)*	*.6791 (0.17)*	*.3029 (1.22)*
			Post	53.86 (1.26)	55.43 (1.26)	55.50 (1.36)			
	Total OP	Amplitude	Pre	239.07 (22.81)	256.30 (22.81)	215.33 (21.84)	*.5504 (0.60)*	***.0299 (4.95)***	*.5289 (0.64)*
			Post	210.92 (17.23)	193.45 (17.23)	195.92 (18.73)			

Supplemental table 7 | Two-way mixed ANOVA results of the scotopic condition at 2.39 cd.s/m^2 of luminance

				Susceptible (M = 2S, F = 14)	Resilient (M=10,F=14)	Control (M=14, F = 13)	Fixed effet		
							Phenotype	*Time*	*Phenotype"Time*
**Sex**	**Wave**	**Parameter**	**Time**	*M (SE)*	*M (SE)*	*M (SE)*	*p (F)*	*p (F)*	*p (F)*
**Male**	a-wave	Amplitude	Pre	-251.74 (13.37)	-247.80 (21.14)	-233.07 (17.86)	.5401 *(0.62)*	***.0104 (6.89)***	*.2887 (1.26)*
***(n* =49)**			Post	-218.42 (9.05)	-188.73 (14.30)	-227.23 (13.06)			
		Implicit time	Pre	17.32 (0.23)	16.80 (0.37)	16.93 (0.31)	.1499 *(1.94)*	*.4666 (0.53)*	***.0120 (4.66)***
			Post	16.88 (0.18)	18.00 (0.28)	16.67 (0.26)			
	6-wave	Amplitude	Pre	547.08 (22.77)	557.50 (36.00)	601.04 (30.42)	*.4298 (0.85)*	***.0161 (6.03)***	*.6209 (0.48)*
			Post	515.39 (17.31)	505.28 (27.37)	521.87 (24.99)			
		Implicit time	Pre	49.80 (0.69)	51.30 (1.09)	50.64 (0.92)	*.2409 (1.45)*	*.8884 (0.02)*	*.2446 (1.43)*
			Post	50.04 (0.76)	49.20 (1.19)	52.17(1.09)			
	Total OP	Amplitude	Pre	320.99 (25.92)	427.83 (43.95)	343.69 (34.48)	*.6535 (0.43)*	.0004 *(13.51)*	***.0058 (5.49)***
			Post	290.60 (21.99)	180.85 (40.71)	317.42 (29.88)			
**Female**	a-wave	Amplitude	Pre	-213.55 (10.85)	-193.71 (10.85)	-213.79(11.72)	.1759 *(1.78)*	***.0000 (19.18)***	*.6288 (0.47)*
***(n* = 41)**			Post	-178.54 (12.93)	-153.34 (12.93)	-155.62 (13.96)			
		Implicit time	Pre	18.64 (0.24)	18.43 (0.24)	18.33 (0.26)	*.1878 (1.72)*	.1215 *(2.47)*	***.0335 (3.59)***
			Post	18.29 (0.39)	18.64 (0.39)	19.75 (0.42)			
	6-wave	Amplitude	Pre	556.74 (27.92)	510.32 (27.92)	534.13 (30.16)	.1491 *(1.95)*	***.0029 (9.47)***	*.9156 (0.09)*
			Post	498.15 (27.62)	435.95 (27.62)	451.96 (29.84)			
		Implicit time	Pre	53.71 (0.77)	51.29 (0.77)	51.67 (0.83)	*.9596 (0.04)*	*.6164 (0.25)*	***.0285 (3.17)***
			Post	50.14 (1.21)	53.07 (1.21)	52.17 (1.31)			
	Total OP	Amplitude	Pre	253.53 (38.21)	218.64 (32.59)	256.72 (38.21)	*.6935 (0.37)*	.2507 *(1.36)*	*.8235 (0.20)*
			Post	215.21 (19.21)	210.83 (20.06)	217.89 (23.53)			

Supplemental table 8 | Two-way mixed ANOVA results of the scotopic condition at 2.86 cd.s/m^2 of luminance

Sex	Wave	Parameter	Time	Susceptible (M = 25, F = 14)	Resilient (M=10,F=14)	Control (M = 14, F = 13)	Fixed effet		
				M (SE)	*M (SE)*	*M (SE)*	*Phenotype* *p (F)*	*Time* *p (F)*	*Phenotype "Time* *p (F)*
**Male (n = 49)**	a-wave	Amplitude	Pre	-301.48 (15.08)	-297.82 (23.85)	-299.47 (20.16)	*.7136 (0.34)*	***.0247 (5.23)***	*.7900 (0.24)*
			Post	-271.67 (10.93)	-250.38 (16.94)	-278.41 (15.46)			
		Implicit time	Pre	15.20 (0.24)	14.70 (0.38)	14.93 (0.32)	*.3199(1.16)*	*.7903 (0.07)*	***.0292 (3.69)***
			Post	14.63 (0.18)	15.60 (0.29)	14.42 (0.26)			
	b-wave	Amplitude	Pre	611.78 (24.65)	596.84 (38.97)	643.61 (32.93)	*.8211 (0.20)*	*.0869 (3.00)*	.6467 *(0.44)*
			Post	567.84 (20.97)	585.57 (32.49)	569.44 (29.66)			
		Implicit time	Pre	49.16 (0.61)	50.10 (0.96)	49.14 (0.82)	*.3140 (1.17)*	.6560 *(0.20)*	.6176 *(0.48)*
			Post	48.25 (0.60)	49.60 (0.93)	49.67 (0.85)			
	Total OP	Amplitude	Pre	362.48 (37.20)	404.89 (56.41)	442.20 (53.79)	*.5319 (0.64)*	***.0402 (4.36)***	*.3992 (0.93)*
			Post	347.84 (29.25)	283.91 (50.67)	346.67 (37.18)			
**Female (n = 41)**	a-wave	Amplitude	Pre	-287.80 (17.49)	-251.12 (16.86)	-256.52 (19.02)	*.1788 (1.16)*	***.0009 (11.95)***	*.8826 (0.13)*
			Post	-230.47 (15.73)	-209.73 (15.73)	-211.22 (16.99)			
		Implicit time	Pre	16.08 (0.26)	16.29 (0.25)	15.82 (0.29)	*.3463 (1.08)*	***.0119 (6.72)***	.1067 *(2.32)*
			Post	16.21 (0.43)	16.79 (0.43)	17.50 (0.46)			
	b-wave	Amplitude	Pre	621.38 (36.44)	560.92 (35.11)	560.94 (39.61)	.1409 *(2.01)*	***.0466 (4.10)***	.9131 *(0.09)*
			Post	560.54 (33.19)	487.63 (33.19)	518.34 (35.85)			
		Implicit time	Pre	SO.92 (0.70)	48.79 (0.67)	49.27 (0.76)	*.7982 (0.23)*	*.9121 (0.01)*	.1746 *(1.79)*
			Post	49.00 (1.02)	50.07 (1.02)	49.67 (1.10)			
	Total OP	Amplitude	Pre	354.56 (59.16)	203.99 (67.08)	336.28 (56.13)	*.0591 (3.05)*	*.1364 (2.31)*	.5580 *(0.59)*
			Post	297.05 (38.65)	193.28 (34.57)	214.82 (41.32)			

Supplemental table 9 | Two-way mixed ANOVA results of the photopic condition at 0.88 cd.s/m^2 of luminance

				Susceptible (M = 25, F = 14)	Resilient (M = 10, F = 14)	Control (M = 14, F = 13)	Fixed effet		
Sex	Wave	Parameter	Time	*M (SE)*	*M (SE)*	*M (SE)*	*Phenotype* *P (F)*	*Time* *P (F)*	*Phenotype*Time* *P (F)*
**Male (*n* = 49)**	a-wave	Amplitude	Pre	-20.92 (1.93)	-26.55 (3.34)	-24.64 (2.73)	***.0786 (2.66)***	*.9581 (0.00)*	*.5402 (0.62)*
			Post	-22.70 (1.81)	-28.24 (2.33)	-21.49 (2.64)			
		Implicit time	Pre	15.33 (0.42)	14.14 (0.67)	14.27 (0.54)	*.2026 (1.64)*	*.9678 (0.00)*	*.3255 (1.14)*
			Post	14.69 (0.35)	14.11 (0.46)	15.00 (0.49)			
	b-wave	Amplitude	Pre	146.32 (9.56)	180.61 (16.56)	156.50 (13.52)	*.2417 (1.46)*	***.0048 (8.64)***	*.3374 (1.11)*
			Post	127.21 (8.18)	129.92 (10.20)	140.44 (11.57)			
		Implicit time	Pre	36.17 (0.54)	36.57 (0.86)	34.82 (0.69)	*.7166 (0.33)*	*.9042 (0.01)*	***.0161 (4.40)***
			Post	35.89 (0.61)	34.44 (0.86)	37.44 (0.86)			
	Total OP	Amplitude	Pre	32.61 (2.80)	36.03 (4.58)	33.18 (3.24)	*.6068 (0.50)*	***.0459 (4.17)***	*.9975 (0.00)*
			Post	27.57 (2.22)	30.54 (2.81)	28.06 (2.57)			
**Female (*n* = 41)**	a-wave	Amplitude	Pre	-19.68 (3.51)	-25.34 (4.30)	-22.98 (4.30)	*.4754 (0.81)*	*.3662 (0.90)*	*.5717 (0.59)*
			Post	-20.65 (3.11)	-22.73 (3.60)	-15.54 (4.40)			
		Implicit time	Pre	14.33 (1.21)	15.67 (1.21)	14.50 (1.48)	*.9331 (0.07)*	*.6656 (0.20)*	*.2732 (1.56)*
			Post	15.67 (0.54)	14.00 (0.67)	16.00 (0.94)			
	b-wave	Amplitude	Pre	133.42 (9.16)	123.29 (11.22)	106.20 (11.22)	*.2694 (1.51)*	*.7999 (0.07)*	*.9462 (0.06)*
			Post	127.00 (12.73)	119.01 (14.70)	108.51 (18.00)			
		Implicit time	Pre	34.20 (1.21)	39.33 (1.56)	37.00 (1.21)	*.2455 (1.53)*	***.0573 (4.16)***	***.0362 (4.08)***
			Post	35.00 (1.01)	33.25 (1.01)	36.00 (1.43)			
	Total OP	Amplitude	Pre	26.19 (2.55)	26.19 (3.13)	28.64 (2.36)	*.9961 (0.00)*	*.7522 (0.11)*	*.9000 (0.11)*
			Post	29.45 (5.14)	28.75 (5.14)	26.96 (8.90)			

Supplemental Tables 9-13. Summary of the two-way mixed model ANOVA results from all recorded densities of light for the photopic condition comparing means and standard errors from susceptible, resilient, and control mice. The tables also display the fixed effect of each interaction from the analysis. *M* = mean; *SE* = standard error, *P* = p-value, *F* = Fisher's statistic. Significance is set at .05.

Supplemental table 10 | Two-way mixed ANOVA results of the photopic condition at 1.37 cd.s/m^2 of luminance

				Susceptible (M=2S,F=14)	Resilient (M=10,F = 14)	Control (M = 14, F = 13)	Fixed effet		
							Phenotype	*Time*	*Phenotype *Time*
**Sex**	**Wave**	**Parameter**	**Time**	*M (SE)*	*M (SE)*	*M (SE)*	*p (F)*	*p (F)*	*p (F)*
**Male**	a-wave	Amplitude	Pre	-34.48 (2.64)	-33.33 (5.76)	-35.03 (3.32)	.6175 *(0.49)*	.1025 *(2.15)*	.4011 *(0.93)*
***(n* =49)**			Post	-29.63 (2.33)	-33.61 (3.94)	-24.49 (3.30)			
		Implicit time	Pre	14.16 (0.41)	13.14 (0.68)	13.58 (0.52)	.1553 *(1.92)*	.6487 *(0.21)*	*.2837 (1.28)*
			Post	13.45 (0.29)	12.71 (0.49)	14.18 (0.39)			
	b-wave	Amplitude	Pre	194.68 (9.15)	211.68 (19.94)	212.46 (11.51)	*.3581 (1.05)*	***.0002 (16.43)***	.6936 *(0.37)*
			Post	165.50 (5.88)	162.97 (10.74)	173.35 (8.32)			
		Implicit time	Pre	34.36 (0.52)	35.00 (0.86)	33.00 (0.70)	*.3717 (1.00)*	.8169 *(0.05)*	***.0034 (6.14)***
			Post	34.86 (0.45)	32.38 (0.74)	35.50 (0.61)			
	Total OP	Amplitude	Pre	50.83 (3.87)	53.09 (6.42)	57.50 (4.85)	*.6802 (0.39)*	***.0019 (10.33)***	.5550 *(0.59)*
			Post	41.30 (2.65)	44.89 (4.11)	40.51 (3.60)			
**Female**	a-wave	Amplitude	Pre	-26.34 (4.56)	-30.22 (5.00)	-22.42 (5.59)	*.8712 (0.14)*	.1475 *(2.30)*	*.5843 (0.55)*
***(n* = 41)**			Post	-31.57 (3.63)	-31.16 (5.13)	-33.80 (4.19)			
		Implicit time	Pre	13.00 (0.42)	14.17 (0.42)	13.25 (0.51)	*.5008 (0.73)*	*.3371 (1.00)*	.4055 *(0.98)*
			Post	13.60 (0.80)	13.67 (1.04)	15.00 (1.04)			
	b-wave	Amplitude	Pre	173.88 (8.59)	173.50 (8.59)	164.78 (9.60)	.1479 *(2.33)*	*.3620 (0.91)*	*.2219 (1.76)*
			Post	187.45 (13.19)	138.90 (18.65)	155.68 (15.23)			
		Implicit time	Pre	33.13 (1.10)	33.40 (1.40)	34.29 (1.18)	*.8277 (0.19)*	.4441 *(0.61)*	.9180 *(0.09)*
			Post	32.80 (1.02)	32.67 (1.31)	33.00 (1.31)			
	Total OP	Amplitude	Pre	35.96 (3.08)	37.65 (3.08)	46.49 (3.30)	*.5229 (0.67)*	*.2040 (1.73)*	***.0413 (3.81)***
			Post	41.82 (3.32)	33.68 (4.28)	33.00 (4.28)			

Supplemental table 11 | Two-way mixed ANOVA results of the photopic condition at 1.89 cd.s/m^2 of luminance

				Susceptible (M = 25, F = 14)	Resilient (M = 10, F = 14)	Control (M=14,F=13)	Fixed effet		
							Phenotype	*Time*	*Phenotype *Time*
**Sex**	**Wave**	**Parameter**	**Time**	*M (SE)*	*M (SE)*	*M (SE)*	*p (F)*	*p (F)*	*p (F)*
**Male (n = 49)**	a-wave	Amplitude	Pre	-35.79 (1.96)	-36.47 (3.31)	-40.08 (2.34)	*.2733 (1.32)*	*.0886 (2.98)*	***.0662 (2.82)***
			Post	-31.75 (1.96)	-39.78 (3.18)	-29.69 (2.71)			
		Implicit time	Pre	12.96 (0.26)	12.25 (0.45)	12.29 (0.34)	.6549 *(0.43)*	*.5641 (0.34)*	*.4084 (0.91)*
			Post	12.22 (0.34)	12.38 (0.51)	12.33 (0.42)			
	b-wave	Amplitude	Pre	238.09 (8.59)	237.64 (14.52)	259.85 (10.27)	*.1429 (2.00)*	**.0000 *(20.17)***	.7695 *(0.26)*
			Post	194.88 (7.62)	206.34 (14.27)	210.34 (10.54)			
		Implicit time	Pre	33.75 (0.53)	33.78 (0.86)	32.36 (0.69)	*.1720 (1.80)*	*.3405 (0.92)*	**.0097 *(4.91)***
			Post	34.43 (0.40)	32.00 (0.68)	34.93 (0.51)			
	Total OP	Amplitude	Pre	77.18 (5.67)	79.98 (9.46)	86.19 (7.58)	*.3666 (1.02)*	**.0000 *(20.64)***	*.7135 (0.34)*
			Post	53.45 (3.09)	63.17 (4.79)	58.14 (4.05)			
**Female (n = 41)**	a-wave	Amplitude	Pre	-31.66 (3.89)	-34.86 (3.67)	-25.42 (4.92)	*.1766 (1.85)*	*.2287 (1.51)*	*.9214 (0.08)*
			Post	-36.35 (2.73)	-37.01 (4.17)	-30.57 (4.17)			
		Implicit time	Pre	13.00 (0.54)	13.50 (0.52)	12.40 (0.73)	*.5160 (0.68)*	*.6445 (0.22)*	*.5821 (0.55)*
			Post	12.71 (0.55)	13.50 (0.73)	13.40 (0.65)			
	b-wave	Amplitude	Pre	198.78 (13.54)	193.14 (12.76)	218.39 (17.12)	*.3031 (1.25)*	*.4083 (0.71)*	*.1971 (1.13)*
			Post	216.19 (10.31)	180.28 (15.75)	184.24 (15.75)			
		Implicit time	Pre	33.78 (1.09)	35.10 (1.04)	34.14 (1.24)	*.9998 (0.00)*	*.4417 (0.61)*	*.5018 (0.70)*
			Post	34.14 (1.12)	32.80 (1.32)	33.80 (1.32)			
	Total OP	Amplitude	Pre	48.32 (5.05)	49.48 (4.79)	62.93 (5.73)	*.4925 (0.72)*	*.1922 (1.17)*	*.1269 (2.19)*
			Post	51.85 (4.23)	46.97 (5.00)	45.69 (5.00)			

Supplemental table 12 **|** Two-way mixed ANOVA results of the photopic condition at 2.39 cd.s/m^2 of luminance

				Susceptible (M = 25, F = 14)	Resilient (M = 10, F = 14)	Control (M=14,F=13)	Fixed effet		
							Phenotype	*Time*	*Phenotype *Time*
**Sex**	**Wave**	**Parameter**	**Time**	*M (SE)*	*M (SE)*	*M (SE)*	*p (F)*	*p (F)*	*p (F)*
**Male (n = 49)**	a-wave	Amplitude	Pre	-41.70 (2.79)	-43.11 (4.51)	-45.69 (3.68)	*.1730 (1.80)*	*.5673 (0.33)*	*.0937 (2.44)*
			Post	-38.93 (2.79)	-50.64 (3.95)	-35.90 (3.46)			
		Implicit time	Pre	11.23 (0.26)	11.44 (0.41)	11.08 (0.34)	*.8495 (0.16)*	*.1877 (1.76)*	*.3827 (0.97)*
			Post	11.09 (0.25)	10.50 (0.37)	11.08 (0.32)			
	b-wave	Amplitude	Pre	273.72 (10.53)	288.24 (17.05)	295.09 (13.92)	.1276 *(2.12)*	**.0000 *(25.06)***	*.9854 (0.01)*
			Post	223.31 (8.00)	241.17 (11.31)	243.55 (9.92)			
		Implicit time	Pre	31.90 (0.50)	31.70 (0.80)	30.80 (0.70)	*.3217 (1.15)*	*.1539 (2.08)*	***.0335 (3.55)***
			Post	32.30 (0.40)	30.80 (0.60)	33.20 (0.50)			
	Total OP	Amplitude	Pre	106.17 (6.50)	110.71 (10.83)	117.46 (8.69)	*.2369 (1.47)*	**.0000 *(27.68)***	*.5393 (0.62)*
			Post	73.17 (3.94)	89.61 (5.98)	78.66 (5.05)			
**Female (n = 41)**	a-wave	Amplitude	Pre	-39.50 (5.80)	-41.45 (5.80)	-32.53 (6.20)	*.9329 (0.07)*	*.1239 (2.50)*	*.2855 (1.31)*
			Post	-41.55 (3.12)	-43.30 (5.84)	-48.83 (3.69)			
		Implicit time	Pre	11.40 (0.48)	11.22 (0.51)	12.14 (0.57)	*.6533 (0.43)*	*.7043 (0.15)*	*.4352 (0.85)*
			Post	11.86 (0.50)	11.20 (0.59)	11.20 (0.59)			
	b-wave	Amplitude	Pre	232.09 (16.83)	239.57 (16.83)	248.71 (17.99)	.7095 *(0.35)*	*.9818 (0.00)*	*.9480 (0.05)*
			Post	237.22 (10.23)	237.46 (19.14)	244.79 (12.11)			
		Implicit time	Pre	31.90 (0.95)	33.20 (0.95)	32.57 (1.14)	*.9442 (0.06)*	*.6351 (0.23)*	*.6394 (0.45)*
			Post	32.43 (0.98)	31.80 (1.15)	32.20 (1.15)			
	Total OP	Amplitude	Pre	71.06 (7.33)	79.28 (7.33)	85.50 (8.76)	*.4104 (0.91)*	*.5421 (0.38)*	.6014 *(0.52)*
			Post	75.23 (5.73)	70.23 (6.78)	79.57 (6.78)			

Supplemental table 13 I Two-way mixed ANOVA results of the photopic condition at 2.86 cd.s/m^2 of luminance

Sex	Wave	Parameter	Time	Susceptible (M = 25, F = 14) M (SE)	Resilient (M = 10, F = 14) M (SE)	Control (M=14,F=13) M (SE)	Fixed effet: Phenotype p (F)	Fixed effet: Time p (F)	Fixed effet: Phenotype *Time p (F)
**Male (n = 49)**	a-wave	Amplitude	Pre	-75.37 (6.38)	-67.62 (10.08)	-80.09 (7.62)	*.1329 (2.07)*	*.3405 (0.92)*	***.0045 (5.79)***
			Post	-64.07 (6.70)	-94.48 (9.71)	-44.92 (8.86)			
		Implicit time	Pre	9.61 (0.26)	10.13 (0.45)	8.93 (0.34)	*.6093 (0.50)*	*.5346 (0.39)*	***.0017 (6.93)***
			Post	9.52 (0.32)	8.30 (0.47)	10.25 (0.43)			
	b-wave	Amplitude	Pre	324.10 (11.83)	335.60 (18.70)	359.28 (14.13)	***.0330 (3.57)***	***.0000 (21.53)***	*.2707 (1.33)*
			Post	261.82 (10.66)	310.48 (15.07)	284.64 (13.76)			
		Implicit time	Pre	30.33 (0.46)	29.88 (0.79)	29.07 (0.60)	***.0703 (2.75)***	*.3259 (0.98)*	***.0126 (4.64)***
			Post	30.68 (0.33)	28.60 (0.48)	31.29 (0.41)			
	Total OP	Amplitude	Pre	133.30 (6.55)	140.40(11.35)	153.68 (8.58)	***.0594 (2.94)***	***.0000 (24.21)***	*.2279 (1.51)*
			Post	103.53 (4.28)	124.48 (6.35)	109.65 (5.36)			
**Female (n = 41)**	a-wave	Amplitude	Pre	-77.5 (10.70)	-79.8 (11.30)	-57.4 (12.80)	*.6610 (0.42)*	*.4539 (0.57)*	*.0788 (2.13)*
			Post	-63.6 (7.60)	-79.6 (10.10)	-90.8 (9.00)			
		Implicit time	Pre	9.00 (0.44)	9.80 (0.44)	8.86 (0.53)	*.4684 (0.78)*	***.0233 (5.65)***	*.2512 (1.44)*
			Post	8.71 (0.20)	8.40 (0.23)	8.40 (0.23)			
	b-wave	Amplitude	Pre	296.11 (22.09)	301.01 (23.29)	304.30 (26.41)	*.6223 (0.48)*	.9068 *(0.01)*	*.7227 (0.33)*
			Post	297.14 (11.67)	281.49 (15.44)	317.14 (13.81)			
		Implicit time	Pre	29.80 (0.91)	31.40 (0.91)	30.86 (1.09)	*.9039 (0.10)*	*.4840 (0.50)*	*.4305 (0.86)*
			Post	30.57 (0.86)	29.80 (1.02)	30.00 (1.02)			
	Total OP	Amplitude	Pre	121.35 (11.23)	124.75 (11.23)	120.01 (13.42)	*.6220 (0.48)*	.6006 *(0.28)*	*.3914 (0.96)*
			Post	113.97 (6.24)	107.78 (8.25)	131.48 (7.38)			

Supplemental table 14 | Estimates and 95% confidence intervals for the scotopic (1.89 log cd.s/m²) and photopic (2.86 log cd.s/m²) conditions in male

				Susceptible (*n* = 25)		Resilient (*n* = 10)		Control (*n* = 14)	
Condition	Wave	Parameter	Time	*M (SE)*	*95% CI*	*M (SE)*	*95% CI*	*M (SE)*	*95% CI*
Scotopic	a-wave	Amplitude (µV)	Pre	-185.7 (10.6)	[-207.1, -164.3]	-196.8 (16.5)	[-229.9, -163.6]	-176.9 (13.9)	[-205.0, -148.9]
			Post	-157.0 (7.3)	[-171.7, -142.3]	-120.7 (11.3)	[-143.5, -97.9]	-170.1 (9.6)	[-189.4, -150.8]
		Implicit time (ms)	Pre	20.3 (0.2)	[19.9, 20.7]	19.8 (0.3)	[19.2, 20.4]	19.6 (0.3)	[19.1, 20.2]
			Post	19.9 (0.2)	[19.5, 20.3]	20.7 (0.3)	[20.1, 21.3]	19.8 (0.2)	[19.3, 20.3]
	b-wave	Amplitude (µV)	Pre	493.0 (21.1)	[450.6, 535.5]	498.4 (32.6)	[432.7, 564.1]	559.3 (27.6)	[503.8, 614.9]
			Post	462.0 (14.6)	[432.6, 491.3]	433.5 (22.5)	[388.1, 478.9]	494.5 (19.1)	[456.1, 532.9]
		Implicit time (ms)	Pre	53.5 (0.5)	[52.4, 54.5]	53.1 (0.8)	[51.5, 54.7]	53.3 (0.7)	[51.9, 54.7]
			Post	53.2 (0.9)	[51.5, 55.0]	50.5 (1.3)	[47.8, 53.2]	53.9 (1.1)	[51.6, 56.2]
	Total OP	Amplitude (µV)	Pre	302.4 (21.0)	[260.0, 344.8]	324.0 (33.6)	[256.3, 391.8]	302.1 (27.9)	[245.7, 358.5]
			Post	281.2 (18.4)	[244.0, 318.4]	183.3 (28.8)	[125.2, 241.4]	315.3 (23.9)	[267.0, 363.7]
Photopic	a-wave	Amplitude (µV)	Pre	-75.4 (6.4)	[-88.3, -62.5]	-67.6 (10.1)	[-88.0, -47.2]	-80.1 (7.6)	[-95.5, -64.7]
			Post	-64.1 (6.7)	[-77.6, -50.5]	-94.5 (9.7)	[-114.1, -74.9]	-44.9 (8.9)	[-62.8, -27.0]
		Implicit time (ms)	Pre	9.6 (0.3)	[9.1, 10.1]	10.1 (0.4)	[9.2, 11.0]	8.9 (0.3)	[8.2, 9.6]
			Post	9.5 (0.3)	[8.9, 10.2]	8.3 (0.5)	[7.4, 9.2]	10.3 (0.4)	[9.4, 11.1]
	b-wave	Amplitude (µV)	Pre	324.1 (11.8)	[300.2, 348.0]	335.6 (18.7)	[297.8, 373.4]	359.3 (14.1)	[330.7, 387.9]
			Post	261.8 (10.7)	[240.3, 283.4]	310.5 (15.1)	[280.0, 341.0]	284.6 (13.8)	[256.8, 312.5]
		Implicit time (ms)	Pre	30.3 (0.5)	[29.4, 31.3]	29.9 (0.8)	[28.3, 31.5]	29.1 (0.6)	[27.9, 30.3]
			Post	30.7 (0.3)	[30.0, 31.3]	28.6 (0.5)	[27.6, 29.6]	31.3 (0.4)	[30.5, 32.1]
	Total OP	Amplitude (µV)	Pre	133.3 (6.6)	[120.1, 146.5]	140.4 (11.3)	[117.5, 163.3]	153.7 (8.6)	[136.4, 171.0]
			Post	103.5 (4.3)	[94.9, 112.2]	124.5 (6.3)	[111.7, 137.3]	109.6 (5.4)	[98.8, 120.5]

Supplemental Table 14. Summary of the estimates and 95% confidence intervals of each ERG parameter in the scotopic condition at 1.89 log cd.s/m^2 and in the photopic condition at 2.86 log cd.s/m^2 during the baseline (pre) and after the stress (post) in all groups of male mice (susceptible, resilient, and control). Confidence intervals were obtained from the LSD multiple comparisons test. *M* = mean; *SE* = standard error, *CI* = confidence interval.
